# Ultrasound and non-ultrasound imaging techniques in the assessment of diaphragmatic dysfunction

**DOI:** 10.1186/s12890-021-01441-6

**Published:** 2021-03-15

**Authors:** Franco A. Laghi, Marina Saad, Hameeda Shaikh

**Affiliations:** 1 Department of Internal Medicine, Sinai Hospital, 2401 W Belvedere Ave, Baltimore, MD 21215 USA; 2 Department of Biomedical and Clinical Sciences (DIBIC), Division of Pulmonary Diseases, University of Milan, Ospedale L. Sacco, ASST Fatebenfratelli-Sacco, V. G.B. Grassi, 74, 20157 Milan, Italy; 3 Division of Pulmonary and Critical Care Medicine, Hines Veterans Affairs Hospital (111N), 5th Avenue and Roosevelt Road, Hines, IL 60141 USA; 4 Division of Pulmonary and Critical Care Medicine, Loyola University Chicago Stritch School of Medicine, 2160 S 1st Ave, Maywood, IL 60153 USA

**Keywords:** Diaphragm dysfunction, Ultrasound imaging, Static imaging techniques, Dynamic imaging techniques, Phrenic nerve, Mechanical ventilation, Neuromuscular disorders

## Abstract

Diaphragm muscle dysfunction is increasingly recognized as an important element of several diseases including neuromuscular disease, chronic obstructive pulmonary disease and diaphragm dysfunction in critically ill patients. Functional evaluation of the diaphragm is challenging. Use of volitional maneuvers to test the diaphragm can be limited by patient effort. Non-volitional tests such as those using neuromuscular stimulation are technically complex, since the muscle itself is relatively inaccessible. As such, there is a growing interest in using imaging techniques to characterize diaphragm muscle dysfunction. Selecting the appropriate imaging technique for a given clinical scenario is a critical step in the evaluation of patients suspected of having diaphragm dysfunction. In this review, we aim to present a detailed analysis of evidence for the use of ultrasound and non-ultrasound imaging techniques in the assessment of diaphragm dysfunction. We highlight the utility of the qualitative information gathered by ultrasound imaging as a means to assess integrity, excursion, thickness, and thickening of the diaphragm. In contrast, quantitative ultrasound analysis of the diaphragm is marred by inherent limitations of this technique, and we provide a detailed examination of these limitations. We evaluate non-ultrasound imaging modalities that apply static techniques (chest radiograph, computerized tomography and magnetic resonance imaging), used to assess muscle position, shape and dimension. We also evaluate non-ultrasound imaging modalities that apply dynamic imaging (fluoroscopy and dynamic magnetic resonance imaging) to assess diaphragm motion. Finally, we critically review the application of each of these techniques in the clinical setting when diaphragm dysfunction is suspected.

**Supplementary Information:**

The online version contains supplementary material available at 10.1186/s12890-021-01441-6.

## Background

The diaphragm is the main muscle of respiration during resting breathing [1]. When respiratory demands are increased or diaphragm function is impaired, rib cage muscles and expiratory muscles are progressively recruited [2]. In some patients with diaphragm dysfunction, this compensation is associated with minimal or no respiratory symptoms [1]. In other patients, this compensation is associated with significant respiratory symptoms. Diaphragm dysfunction can cause alveolar hypoventilation and, in the most severe cases, respiratory failure requiring mechanical ventilation [1, 2]. The ultimate causes of diaphragmatic dysfunction can be broadly grouped into three major categories: disorders of central nervous system or peripheral neurons, disorders of the neuromuscular junction and disorders of the contractile machinery of the diaphragm itself [3] (Table 1). Early diagnosis of diaphragmatic dysfunction is essential, because it may be responsive to therapeutic intervention [4–7].

**Table 1:** Pathophysiological causes of diaphragmatic dysfunction

Group	Examples
Neuronal disorders	
a) Disorders of CNS	Poliomyelitis, ALS, multiple sclerosis, spinal cord injury
b) Disorders of peripheral neurons	Guillain–Barre syndrome, chronic inflammatory demyelinating polyneuropathy, critical illness polyneuropathy, compression of the phrenic nerve by neighboring structures
Disorders of the neuromuscular junction	Myasthenia gravis, Lambert-Eaton syndrome, botulism, organophosphates
Disorders of the muscle	Muscular dystrophies, critical-illness myopathy, acid maltase deficiency (Pompe disease), dermatomyositis

*CNS* central nervous system, *ALS* amyotrophic lateral sclerosis

A number of static and dynamic imaging techniques are used in the evaluation of patients suspected of diaphragm dysfunction [8]. Static imaging techniques are used to assess the position, shape and dimensions of the diaphragm and include chest radiography [9], brightness mode (B-mode) ultrasound [10, 11], computed tomography (CT) [12], and static magnetic resonance imaging (MRI) [13]. Dynamic imaging techniques are used to assess diaphragm motion in one or more directions. This group of imaging techniques include fluoroscopy [14], motion mode (M-mode) ultrasonography [10, 11, 15], and dynamic MRI [16].

The purpose of this review is to present the accumulated knowledge on imaging techniques used in the evaluation of diaphragm dysfunction. A MEDLINE search of articles published between 2010 and 2020 was undertaken. Searches of the bibliographies of articles resulted in several additional articles and book chapters. Information was selected on the basis of scientific quality and potential relevance to patients suffering from a pulmonary disease or a disorder requiring admission to an intensive care unit; in all, a total of 128 sources were included in this review.

In this review we will first discuss ultrasound imaging techniques and then non-ultrasound imaging techniques. We will then discuss the clinical applications of these techniques. Finally, we will provide a critical appraisal of the limitations of ultrasound and non-ultrasound imaging in the evaluation of diaphragmatic dysfunction and considerations about future directions.

### Ultrasound imaging of the diaphragm

**Fig. 1:**
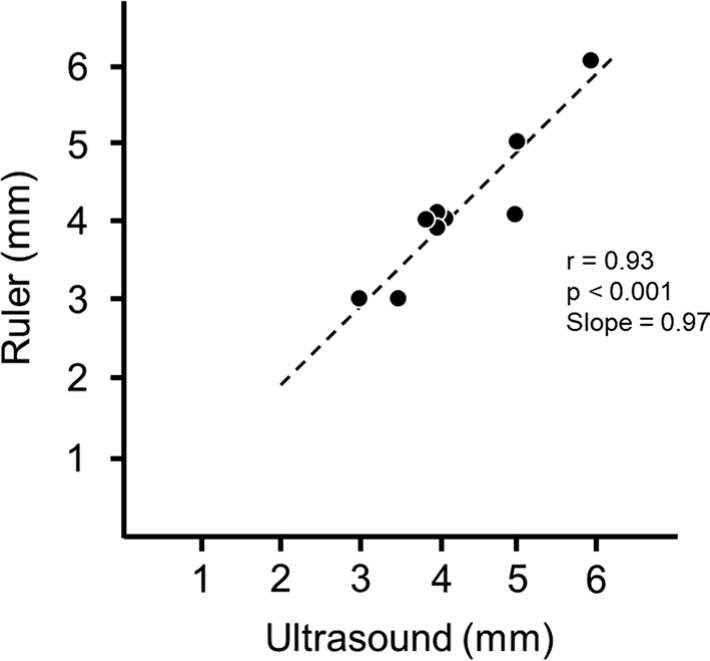
Relationship of diaphragm thickness measured in situ in 10 human cadavers by ultrasound and in vitro by a ruler. Ultrasound measurements of diaphragm thickness in situ are as accurate as measurements in vitro with a ruler. Reproduced with permission from The American Physiological Society: Wait et al. J Appl Physiol 1989;67(4):1560–1568

Diaphragm ultrasonography was first described in the late 1960s as a means to determine position and size of supra- and subphrenic mass lesions, and to assess the motion and contour of the diaphragm [17]. Two decades later, Wait et al. [18] developed a technique to measure diaphragm thickness based on ultrasonography. The investigators reported a close correlation between diaphragm thickness measured in cadavers using ultrasound imaging and thickness measured with a ruler (Fig. 1). Since the seminal work of Wait et al. [18], investigators have published a growing number of studies on the use of ultrasonography to monitor the thickness of the diaphragm in the zone of apposition, the motion of the dome and to estimate diaphragm strength and recruitment during voluntary contractions [19, 20].

#### Ultrasound measurement of diaphragm thickness (zone of apposition)

**Fig. 2:**
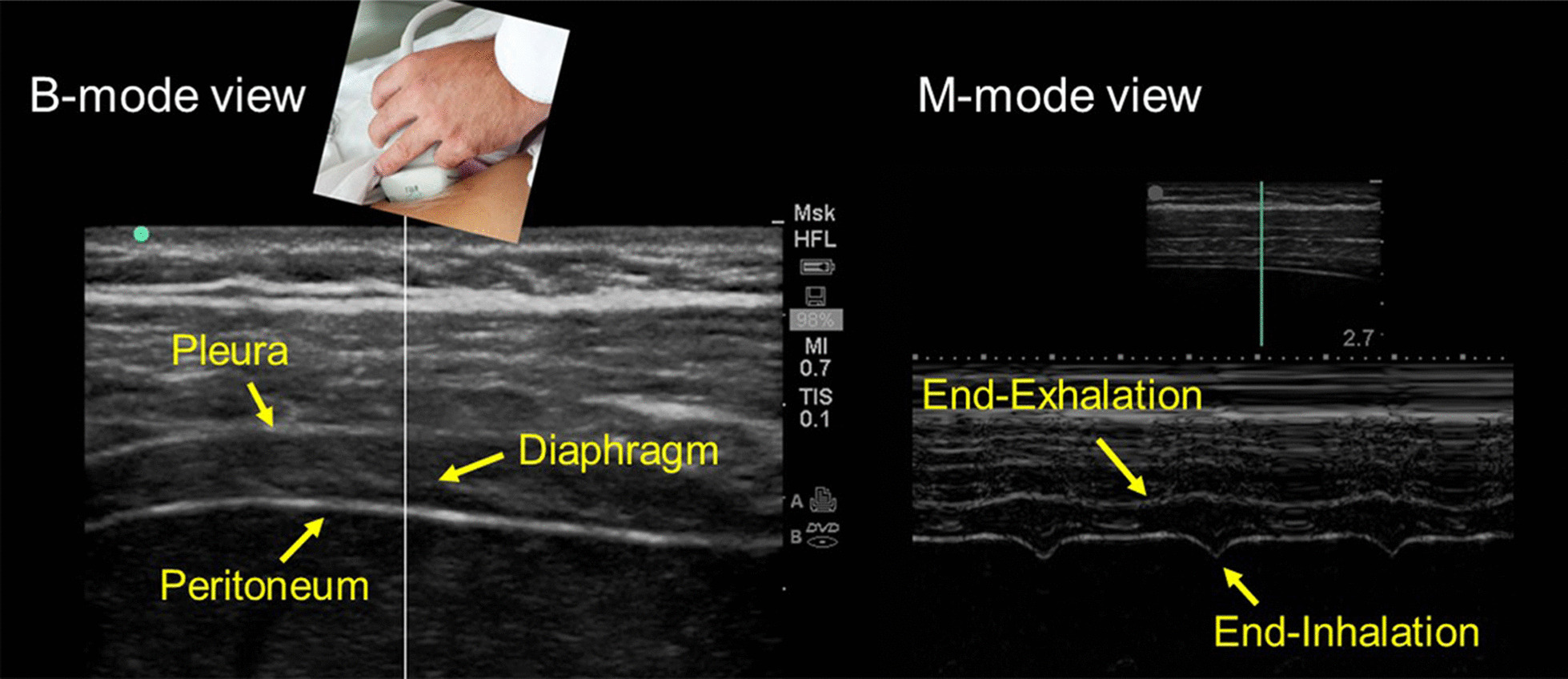
Ultrasound image of the zone of apposition of the diaphragm. In brightness-mode (B-mode; *left panel*), the diaphragm appears as a three-layer structure. In motion-mode (M-mode; *right panel*), the diaphragm is thinnest at end-exhalation and thickest at end-inhalation; stronger diaphragmatic inspiratory efforts are associated with greater tidal thickening of the diaphragm. Reproduced with permission from The American Association for Respiratory Care: Shaikh et al. Respir Care 2019;64:1600–2. The entirety of both images was obtained by Dr. Shaikh

Operators use linear ultrasound probes to measure diaphragm thickness [15] (Fig. 2). These probes use high frequency ultrasound waves (7–18 Hz) to create high resolution images of structures near the body surface [15]. To measure diaphragm thickness, operators place the ultrasound probe longitudinally parallel to the long axis of the body, usually between the eighth to tenth intercostal space [21, 22], at the anterior axillary line [15] or midway between the anterior- and mid-axillary lines [23]. The costo-phrenic sinus [24] is identified as the transition between lung and liver (right) or between lung and spleen (left). The zone of apposition, where the diaphragm is opposed to the rib cage, is located caudal to the costo-phrenic sinus [24]. To identify the diaphragm, subjects are asked to inhale while operators select B-mode imaging. As the lung comes between transducer and diaphragm, it creates an hyperechoic bright artifact (“lung curtain sign”) [25] that obliterates the muscle’s image [18] (Additional file 1). The diaphragm is identified as a three-layer structure (two echogenic layers of peritoneum and pleura sandwiching a more hypoechoic layer of the muscle itself) underneath the intercostal muscles [26] that reappear as lung artifact recedes [15, 18] (Fig. 2). Occasionally, an additional bright layer due to connective tissue and vessels can be seen within the muscle layer itself [27]. To reduce lung artifact operators can move the transducer towards the anterior axillary line [25] or to the next (caudal) intercostal space. It is easier to visualize the right than the left hemidiaphragm [24, 28].

Intra- and interobserver agreement of measurements of diaphragm thickness obtained at a single sitting in healthy adults [26, 29] and in ventilated patients [28] are high as long as the operator marks the site and all subsequent images are recorded from that mark [28]. This caveat is critical because measurements of diaphragm thickness are highly variable depending on the chosen intercostal space: in a study of 150 healthy subjects [26] investigators reported as much as a 6-mm change in resting thickness from one intercostal space to another.

Diaphragm thickness while healthy subjects rest at functional residual capacity (FRC) varies widely from 1.2 to 11.8 mm among individuals, with group mean values ranging from 1.6 to 3.4 mm [18, 25, 26, 29–32]. The lower limit of normal (LLN) in adults can range from 0.80 to 1.60 mm [26, 29–31]. Some of these wide variations may be due to failure to standardize body position [33], lung volume or the intercostal space where the thickness of the diaphragm is measured. The thickness of the diaphragm varies, with more inferior portions of the diaphragm being thicker than more superior portions [15]. Measurements of diaphragm thickness can be difficult in some individuals: a poor acoustic window occurs in 2–10% of ambulatory subjects [34, 35] and 5–15% of intensive care unit (ICU) patients [28, 34]. Adiposity has a detrimental effect on the quality of ultrasound imaging [27].

#### Ultrasound estimation of diaphragm strength and recruitment (zone of apposition)

Recordings of diaphragm thickening during voluntary contractions, two-dimensional speckle tracking imaging and shear wave elastography are ultrasound-based techniques that have been used to estimate diaphragm strength.

##### Diaphragm thickening

**Fig. 3:**
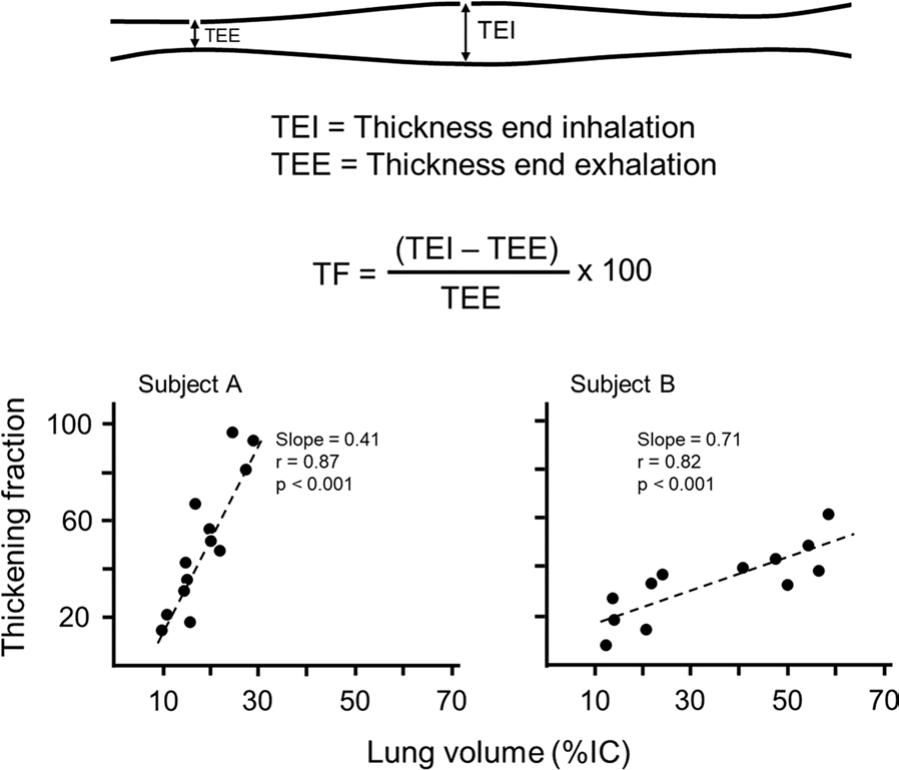
Diaphragmatic thickening fraction. *(Upper panel*) Schematic representation of the points used to measure diaphragm thickness and formula used to calculate thickening fraction (TF). *(Lower panels)* Relationship of thickening fraction to lung volume expressed as a percent of inspiratory capacity (IC) in two healthy subjects. Each point represents the mean of three measurements taken from one breath. The slope of the relationship between thickening fraction and lung volume has high interindividual variability. Reproduced with permission from The American Physiological Society: Wait et al. J Appl Physiol 1989;67(4):1560–1568

**Fig. 4:**
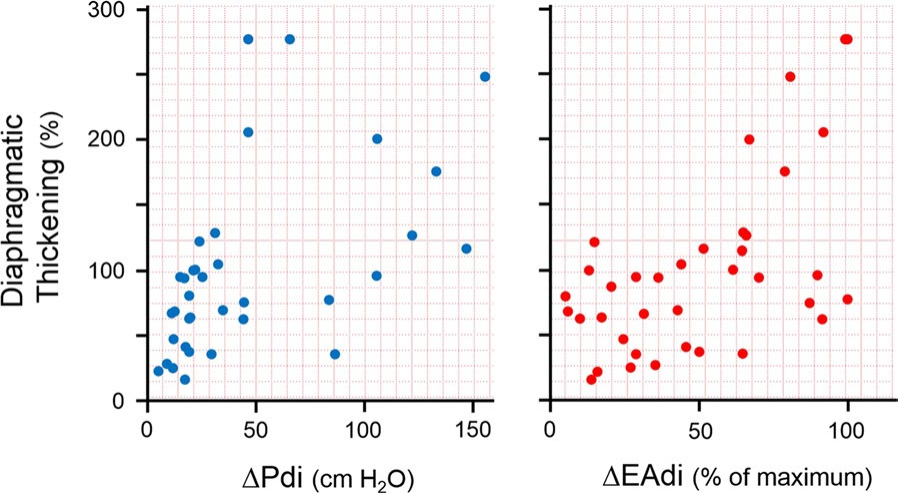
Relationship between the thickening of the diaphragm recorded with ultrasonography and changes in transdiaphragmatic pressure (∆Pdi; *left panel*) and diaphragmatic electrical activity (∆EAdi; *right panel*) in five healthy subjects during a series of inspiratory maneuvers. Diaphragmatic thickening increased as transdiaphragmatic pressure (*left panel*) and electrical activity of the diaphragm (*right panel*) increased. The correlation was weak (r^2^ = 0.32 and 0.28, respectively, p < 0.01). Adapted with permission from Springer Nature: Goligher et al. Intensive Car Med 2015;41(4):642–9

The contracting diaphragm shortens and thickens [18]. This thickening can be quantified as *thickening fraction* (change in thickness from end exhalation to peak inhalation divided by thickness at end exhalation × 100 [18]) (Fig. 3) or as *thickening ratio* (thickness at peak inhalation divided by thickness at end exhalation [27]). According to some [27, 28] but not all investigators [36], diaphragm thickening during voluntary contractions correlates with inspiratory pressures [27, 28] (Fig. 4). When a correlation has been reported, there is high inter-individual variability in the relationship between diaphragmatic thickening and changes in airway pressure (Paw) [27], transdiaphragmatic pressure (Pdi) or electrical activity of the diaphragm (EAdi) [28]. A likely contributor for the high inter-individual variability is shifting recruitment of the various inspiratory muscles during inspiration [37].

##### Two-dimensional speckle tracking imaging

**Fig. 5:**
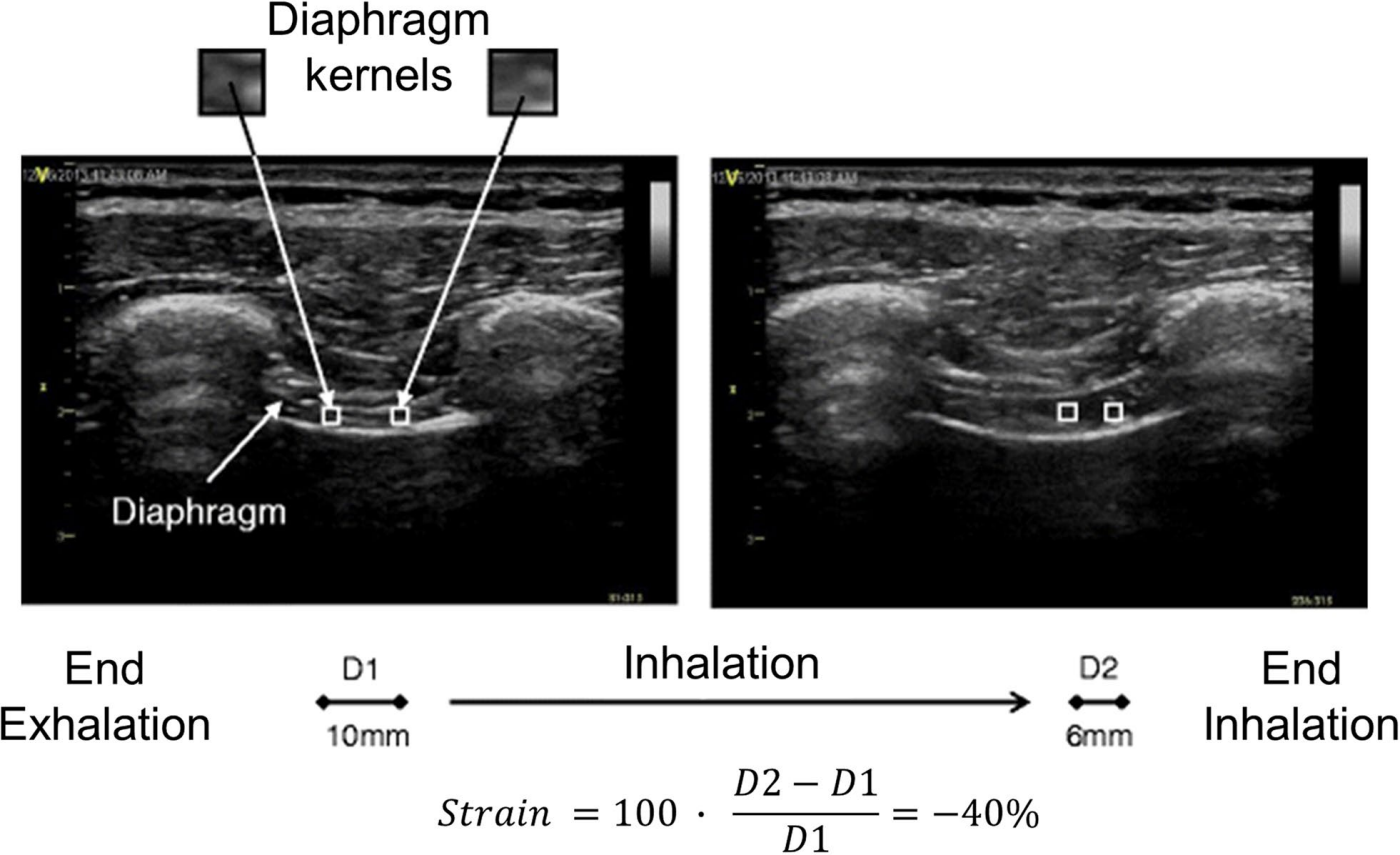
Two-dimensional speckle tracking imaging of the diaphragm’s zone of apposition at end-exhalation (*left panel*) and end-inhalation (*right panel*). The image of the diaphragm has a granularity caused by an inherent ultrasound artifact known as speckle. A cluster of speckles form a kernel. The stronger the contraction of the diaphragm, the closer kernels come together (strain). With speckle-tracking software, it is possible to quantify the strain of the diaphragm as: 100 multiplied by the difference of the distance between two representative kernels at end-inhalation (D2) minus the distance between the same kernels at end-exhalation (D1) divided by D1. In the example above, the distance between two representative kernels at end-exhalation (*left panel*) is 10 mm (D1) and at end-inhalation (*right panel*) 6 mm (D2), yielding a strain of -40%. Reproduced under Open Access Creative Commons License: Orde et al. BMC Anesthesiol 2015;16(1):43

The measurement of diaphragm thickening does not assess contraction-associated longitudinal muscle shortening—the plane of muscle fiber motion [25]. Speckle tracking has the potential to describe this longitudinal shortening during diaphragm contractions [25, 36]. This technique takes advantage of the fact that ultrasound images are made up of different grey-scale pixels called speckles. A speckle-tracking software follows unique groups of these pixels (known as ‘kernels’) to measure their displacement in relation to one another (deformation) [36, 38] (Fig. 5). The extent of deformation is known as ‘strain’. Negative strain values indicate kernels are coming closer together (Additional file 2). For example, a strain value of -30% indicates local muscle fiber shortening of 30%. The more negative a number, the greater the deformation and the greater the contraction (Fig. 5). During loaded breathing, strain is closely correlated with Pdi (*r*^2^ = 0.72) and EAdi (*r*^2^ = 0.60), whereas diaphragmatic thickening is not [36].  The performance of this technique under different loading conditions (isometric contractions, high inspiratory volume) is still unknown.

##### Shear wave elastography

Ultrasound shear wave elastography is an imaging method that allows real-time quantification of tissue mechanical properties [39]. Shear wave elastography relies on the estimation of the propagation velocity of shear waves generated inside tissues [39]. With this technique it is possible to calculate the *shear modulus* (SM) of the tissue being studied [40]. (The *shear modulus,* or *modulus of rigidity*, is defined as the ratio of shear stress to the shear strain where shear stress refers to the deforming force applied on an object, and shear strain refers to the change in size or shape that object.) In limb muscles, local muscle stiffness measured using shear wave elastography provides estimates of muscle force [41].

Chino et al. [42] were the first to report that the diaphragm’s shear modulus (SMdi) increases along with increases in Paw. The rate of increase of the shear module slowed when the pressure reached higher levels. In a study by Bachasson et al. [22] investigators determined whether shear wave elastography could be used as a surrogate of Pdi in healthy subjects. In that study, mean Pdi was related to mean SMdi during closed-airways maneuvers and during inspiratory threshold loading (Additional file 3). The intra- and inter-rater agreement of SMdi measurements are yet to be determined.

#### Ultrasound measurement of diaphragm motion (dome)

**Fig. 6:**
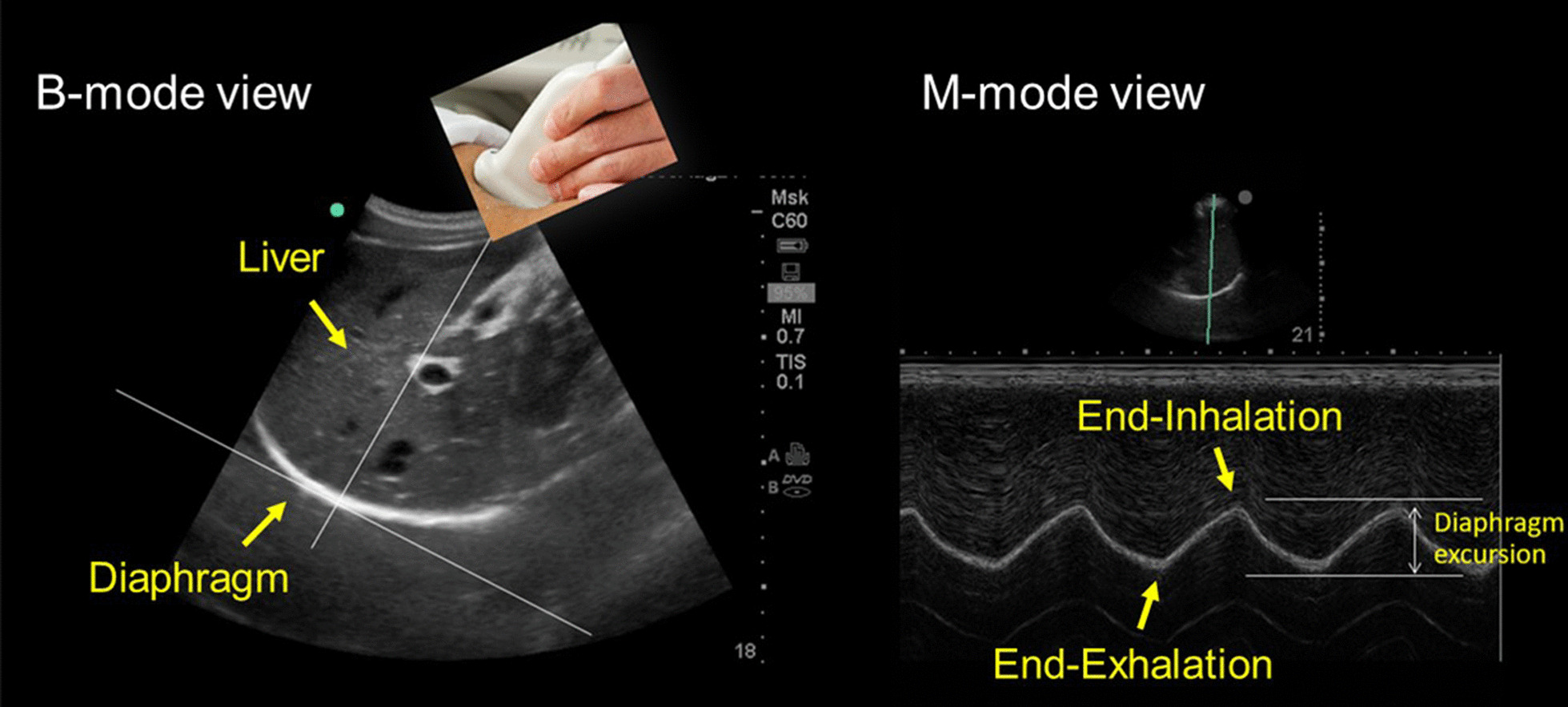
Ultrasound image of the dome of the diaphragm in brightness-mode (B-mode; *left panel*) and motion-mode (M-mode; *right panel*). As the diaphragm contracts, the dome moves towards the ultrasound probe. The larger the caudal displacement of the diaphragm, the greater the diaphragmatic contribution to tidal breathing. Reproduced with permission from The American Association for Respiratory Care: Shaikh e al. Respir Care 2019;64:1600–2. The entirety of both images was obtained by Dr. Shaikh

The cranio-caudal movement of the dome of the diaphragm during quiet breathing [31, 43–45] and during forceful inspiratory efforts such as sniff maneuvers or maximal inspirations [43, 45, 46] can be monitored using curvilinear ultrasound probes. Curvilinear probes use low frequency ultrasound waves (2–6 Hz) [15] that penetrate deeply in the body giving a wide depth of field. On the right, operators position the probe longitudinally in the subcostal area between the mid-clavicular and anterior axillary lines using the liver as acoustic window. The probe is directed medially, cephalad and dorsally so that the ultrasound beam reaches the right dome of the diaphragm perpendicularly. On the left side, operators use the spleen as an acoustic window [45]. (Less often, operators may use the right or left lateral view (midaxillary lines) [47] or the posterior subcostal view or the subxiphoid view [15].) Once a good quality B-mode image is obtained, operators adjust the M-mode interrogation line as to be perpendicular to the movement of the hemidiaphragm [45, 48]. With M-mode ultrasonography, the diaphragm appears as a single thick echogenic line (Fig. 6). During inhalation the contracting diaphragm moves towards the ultrasound probe (Additional file 4). Diaphragm excursion are greater in men than in women [43, 45, 46, 49]. In up to 28% of patients, it is impossible to record maximal diaphragmatic excursions with M-mode ultrasonography [50].

Reported normal values of diaphragm motion during quiet breathing and deep breathing range from 2.6 to 30 mm [43, 45, 46], and 16.7 to 110.0 mm [43, 45, 46], respectively. Diaphragm motion is greater posteriorly than anteriorly and greater laterally than medially [46]. The reported LLN of diaphragm excursion can range from 6.8 to 9.1 mm during resting breathing and from 29.3 to 61.8 mm during deep breathing [31, 43]. Some of these wide variations may be due to failure to standardize body position [13, 14], lung volume, and gender distribution among study participants [44, 45].

The association between diaphragm excursion and diaphragm thickening is very weak [25] and that between diaphragm excursions and diaphragm pressure output [31, 51] is weak-to-absent. A likely contributor for the limited [31] or absent [51] correlation between inspiratory pressure output and diaphragm excursions is the high inter-individual variability in recruitment of various inspiratory muscles during inspiration [37].

### Non-ultrasound imaging of the diaphragm: static imaging techniques

#### Chest radiography

**Fig. 7:**
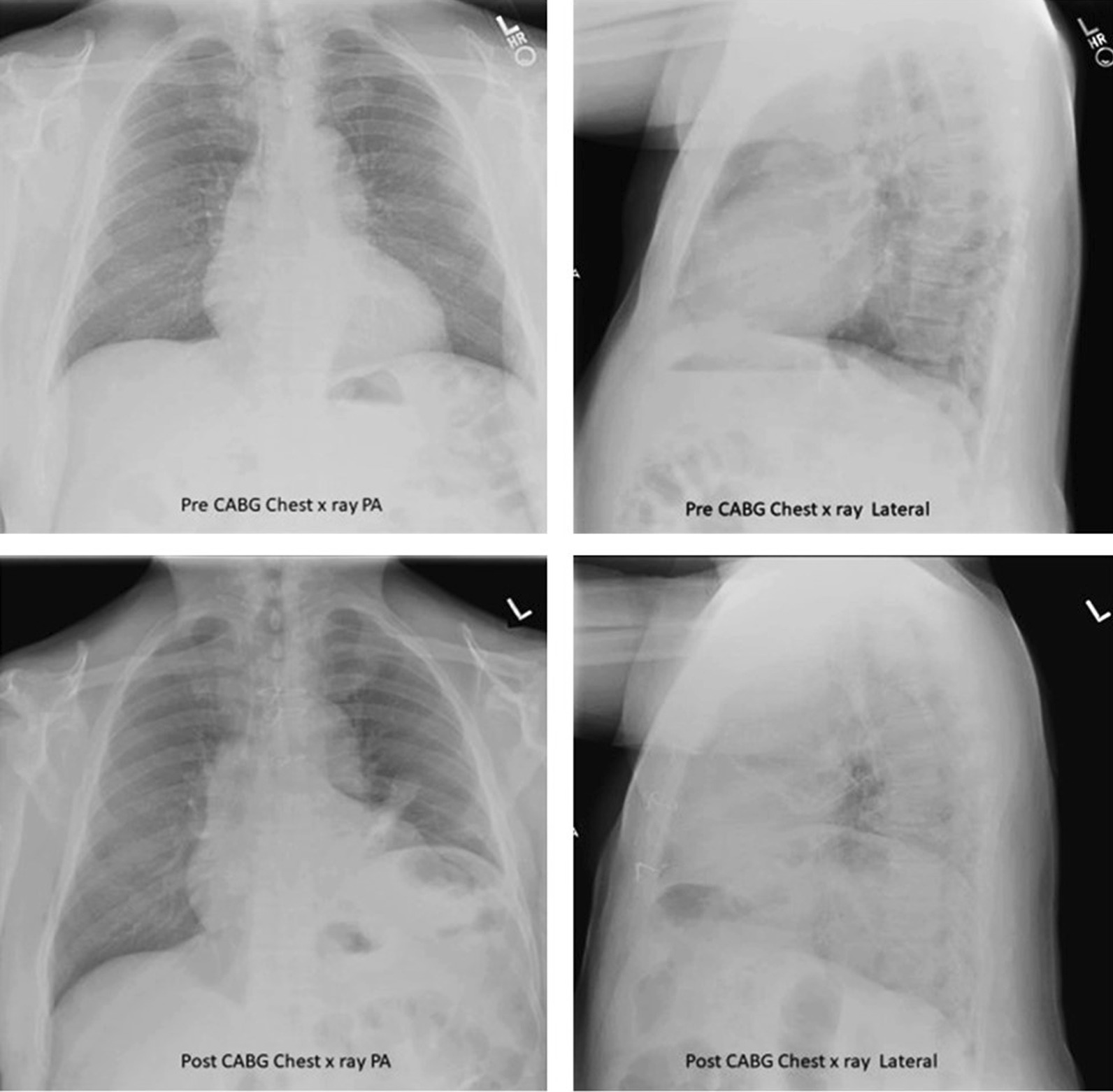
Phrenic nerve injury and diaphragm dysfunction in a patient after coronary artery bypass surgery (CABG). *(Top panels)* Initial posteroanterior (left) and lateral (right) films demonstrate both hemidiaphragms in a relatively normal position. *(Bottom panels)* After CABG, posteroanterior (left) and lateral (right) films demonstrates new elevation of the left hemidiaphragm, suggestive of postoperative phrenic nerve injury. Reproduced under Open Access Creative Commons License: Kokatnur et al. Diseases 2018:6(1):16

**Fig. 8:**
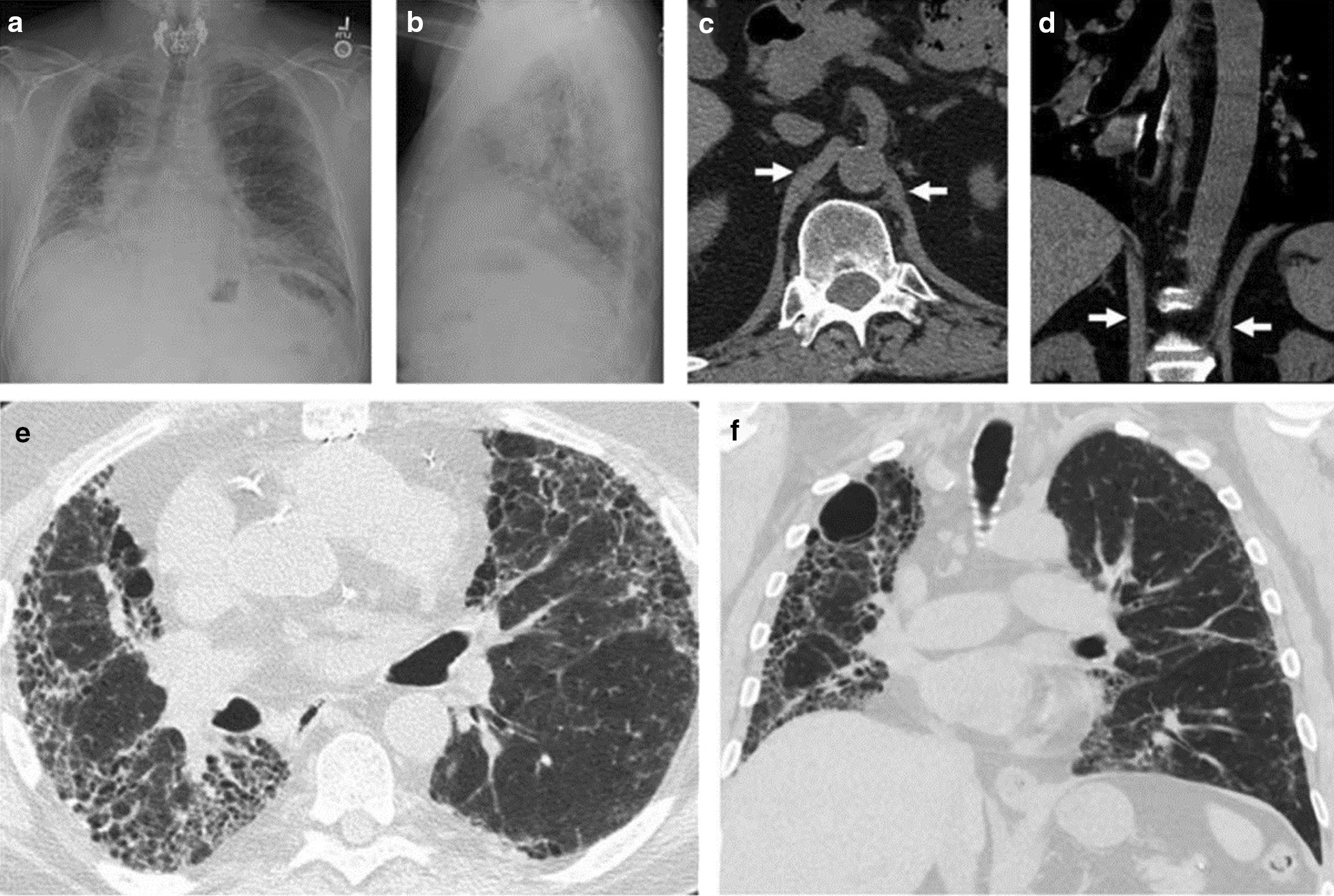
Right hemidiaphragm elevation in a patient with idiopathic pulmonary fibrosis and dyspnea. Posteroanterior (**a**) and lateral (**b**) radiographs show pulmonary fibrosis with low lung volume and elevation of the right hemidiaphragm, concerning for paralysis. Axial (**c**) and coronal (**d**) CT images demonstrate diaphragm crura (arrows) without thinning, arguing against paralysis. CT images with lung window views in the axial (**e**) and coronal (**f**) plane show more lung fibrosis on the right than on the left. Right hemidiaphragm elevation likely reflects the greater degree of fibrosis and volume loss on the right. Reproduced with permission from Wolters Kluwer Health: Sukkasem et al. Journal of Thoracic Imaging 2017;32(6):383–390

Chest radiography is used to assess the position of each hemidiaphragm. An elevated hemidiaphragm suggests unilateral phrenic nerve paralysis (Fig. 7). This, however, is a nonspecific finding that can occur in several other conditions including atelectasis, pneumonia, lobectomy and pulmonary fibrosis (Fig. 8). Another limiting factor of chest radiography is the moderate interobserver agreement to detect the presence of unilateral hemidiaphragm elevation (kappa value ranging from 0.48 to 0.59) [52]. It can be difficult to detect diaphragm elevation in patients with bilateral paralysis unless chest radiographs before the onset of the paralysis are available for comparison [53].

#### Computed tomography

CT images obtained while subjects maintain different lung volumes have been used to assess diaphragm position [54], and diaphragm dimensions in terms of thickness [55–57], surface area [58] and volume [59].

**Fig. 9:**
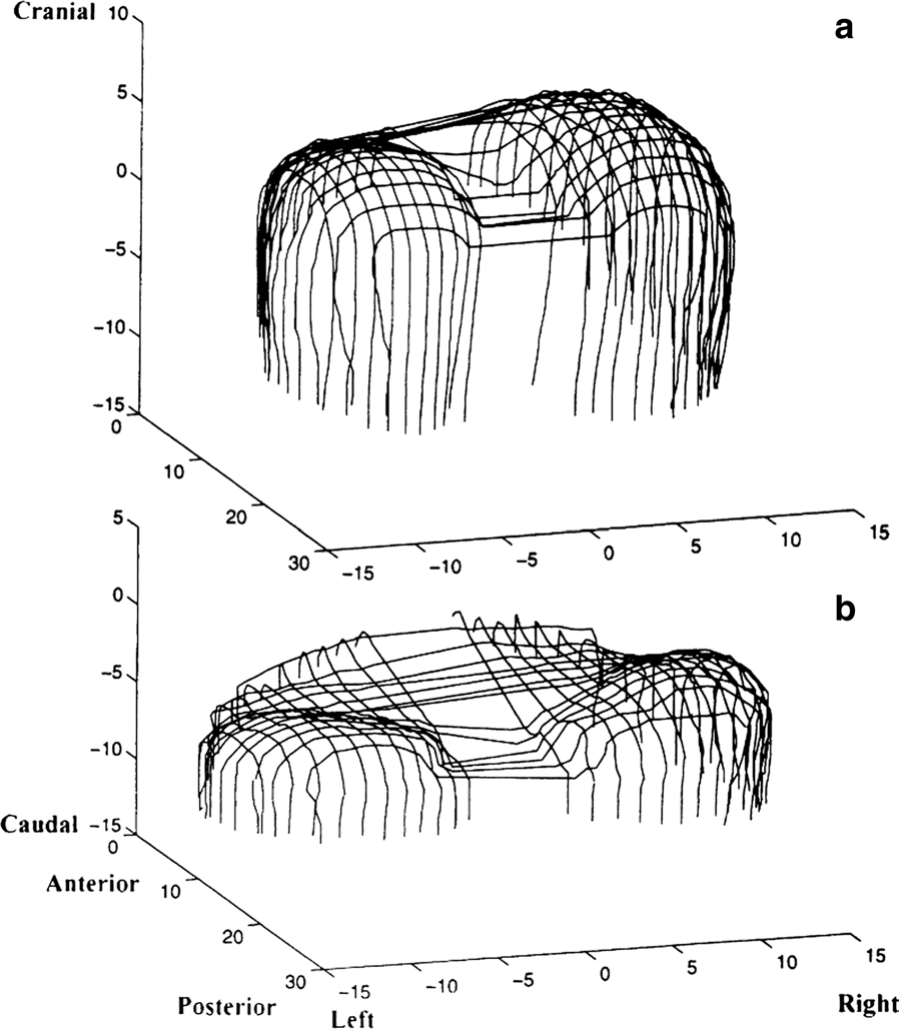
Three-dimensional reconstructed images of the diaphragm at functional residual capacity in (**a**) a control subject and (**b**) a patient with COPD. The numerical units represent centimeters. Compared to the control subject, the patient with COPD has a markedly smaller muscle surface area. Reproduced with permission from The American Thoracic Society: Cassart et al. Am J Respir Crit Care Med 1997;156:504–508

In 1987, Whitelaw [60] was the first to generate a three-dimensional reconstruction of the diaphragm in one healthy subject using serial CT images. Ten years later, Pettiaux et al. [61] validated a technique of using spiral CT in four healthy subjects. Using this technique, Cassart et al. [58] studied the effect of chronic hyperinflation on diaphragm length and surface area in 10 patients with severe chronic obstructive pulmonary disease (COPD) (forced expiratory flow in one second (FEV_1_) = 27 ± 6% (S.D.) predicted) with severe hyperinflation (functional residual capacity (FRC) = 225 ± 2% predicted) and 10 healthy subjects matched for age, sex, and height (Fig. 9). They concluded that patients with COPD have marked reductions in the diaphragm’s total surface area and surface area of the zone of apposition at FRC. At similar absolute lung volumes, however, diaphragm dimensions of patients were similar to those of healthy subjects.

**Fig. 10:**
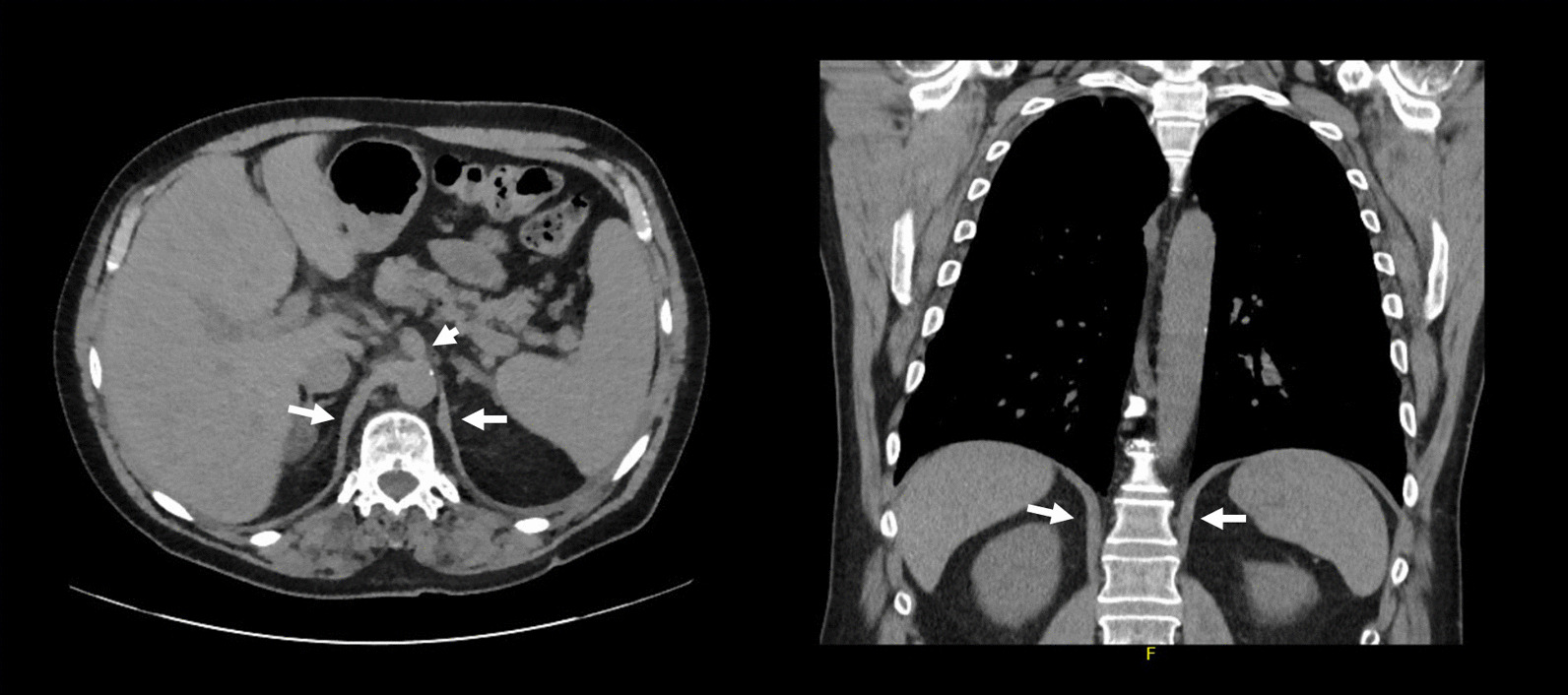
Assessment of crural diaphragm thickness by chest CT using axial and coronal images. (*Left panel*) With axial imaging, the thickness of the right and left crural hemidiaphragms (arrows) can be measured at level of the origin of the celiac artery (arrowhead). Note the nodularity of the left crus, a normal variant of the diaphragm’s shape. (*Right panel*) With coronal imaging, the thickness of the crural hemidiaphragms (arrows) can be measured at level of the first lumbar vertebra

CT imaging has been proposed as a means to measure crural diaphragm thickness in ventilated patients [55] (Fig. 10) and in patients with suspected diaphragm paralysis [56]. Unfortunately, there is no consensus on which area of the muscle should be measured and at which lung volume [57]. Accordingly, the role of CT measurement of the crural diaphragm thickness remains uncertain.

**Fig. 11:**
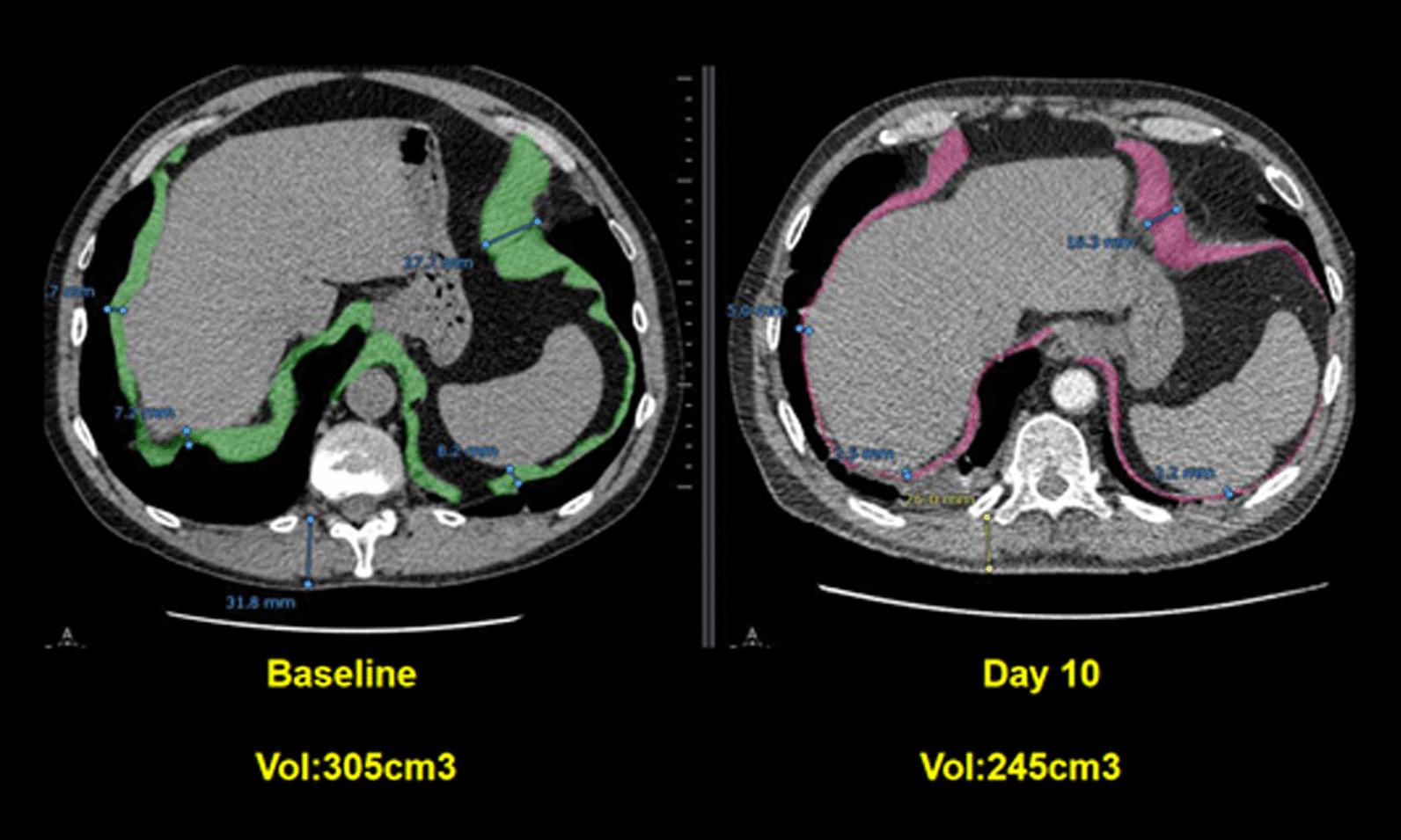
Computed tomography measurements of diaphragm volume in a critically ill, septic patient on admission to the intensive care unit (*left panel*) and ten days later (*right panel*). Sepsis was associated with a decrease in diaphragm volume. The images were provided, with permission, by Drs. Boris Jung, Stephanie Nougaret and Samir Jaber, University Hospital of Montpellier, France

Spiral CT has been used to calculate the volume of the diaphragm [59]. With this technique, Jung et al. [59] reported that, upon ICU admission, the diaphragm’s volume in 23 critically ill patients (14 of whom were septic) was not different from the volume of the diaphragm in 17 control patients. Twenty-five days after admission, the volume of the diaphragm had decreased by 11 ± 13% in nonseptic patients and by 27 ± 12% in septic patients (p = 0.01) (Fig. 11). Upon ICU admission, diaphragm volume only weakly correlated with diaphragm strength as measured by airway twitch pressure (PawTw) elicited by magnetic stimulation of the phrenic nerves. To date, no investigator has validated the accuracy of spiral CT imaging to calculate the volume of the diaphragm.

#### Static magnetic resonance imaging

Static MRI obtained while subjects maintain different lung volumes can be used to assess the diaphragm’s shape [62], position [63], thickness [54] and surface area [58, 64].

Using static MRI in four healthy subjects, Paiva et al. [65] concluded that the shape of the dorsal half of the relaxed diaphragm in the supine position at FRC can be explained by the Laplace law—i.e., the lung and abdominal contents do not impose their shape on the diaphragm. In a subsequent static MRI study, Gauthier et al. [64] reported that, with lung inflation, the dimension of the zone of apposition is a function of diaphragm shortening and, to a lesser extent, widening of the lower rib cage. The investigators concluded that the diaphragm is more accurately modeled by a "widening piston" (Petroll's model) than a simple "piston in a cylinder" model.

**Fig. 12:**
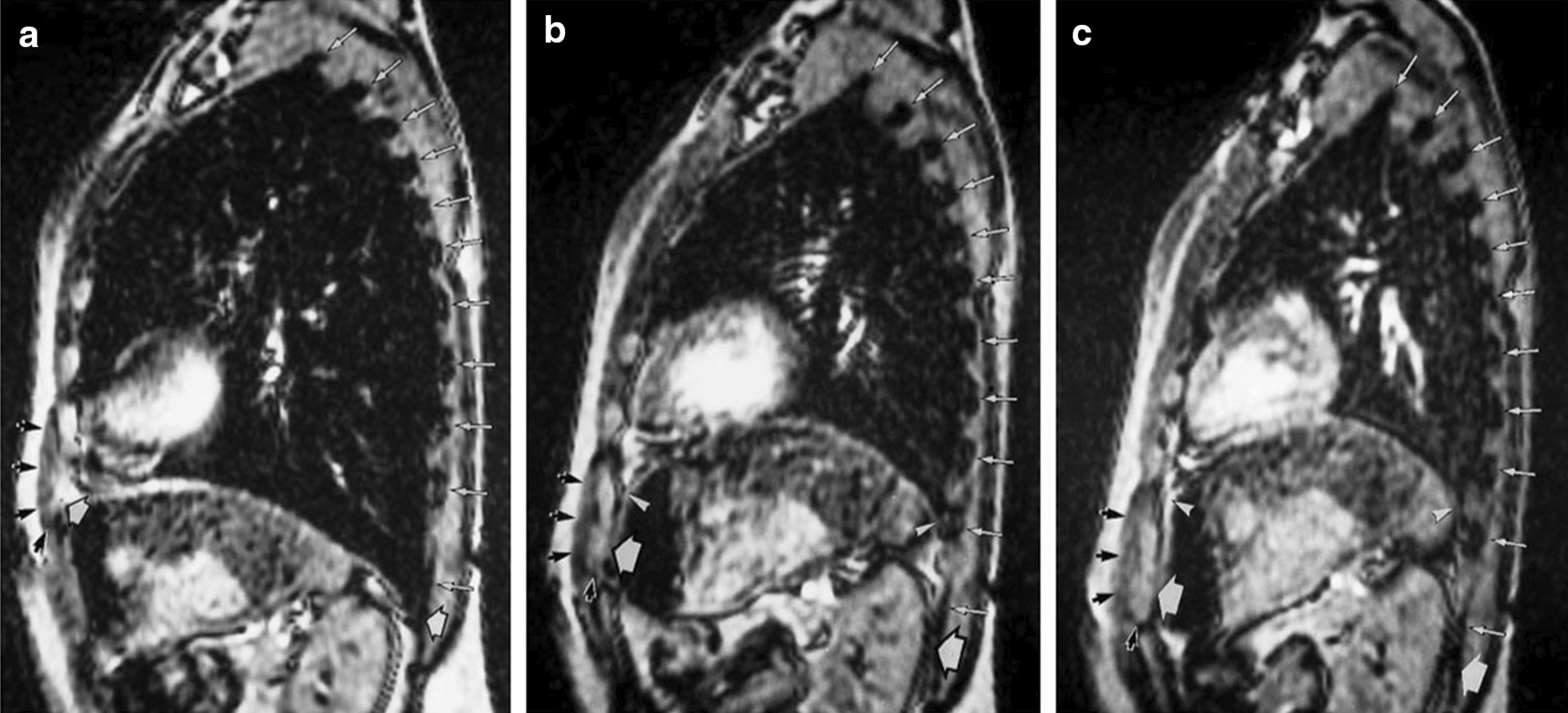
Magnetic resonance images of the diaphragm and rib cage in the sagittal view. Images were obtained at (**a**) total lung capacity, (**b**) functional residual capacity, and (**c**) residual volume, 7 cm to the left of the midline. At total lung capacity (**a**) the anterior insertion of the diaphragm onto chest wall (left white arrowhead) is in close proximity to the lower anterior ribs (black arrows) indicating that the zone of apposition is close to zero. Posteriorly, the diaphragm is positioned nearly at the level of the posterior insertion point at the twelfth rib (right white arrowhead). As the diaphragm moves to functional residual capacity (**b**) and residual volume (**c**) the zone of apposition increases indicated by the cranial movement of the upper limits of the zone of apposition (right and left thin white arrowheads). Reproduced with permission from Radiologic Society of North America: Cluzel et al. Radiology 2000;215(2):574–83

More recently, Cluzel et al. [62] evaluated the use of three-dimensional reconstruction of the static MRI of the thorax in five healthy subjects while supine. Compared to measurements of residual volume (RV) and total lung capacity (TLC) obtained using a spirometer and helium dilution technique, measurements obtained with static MRI tended to overestimate RV and underestimate TLC. Between RV and TLC, the mean volume under the dome of the diaphragm decreased by 66%, and the mean volume of the cavity delimited by the rib cage increased by 23%. The diaphragm contributed to 60% of the inspiratory capacity (Fig. 12).

**Fig. 13:**
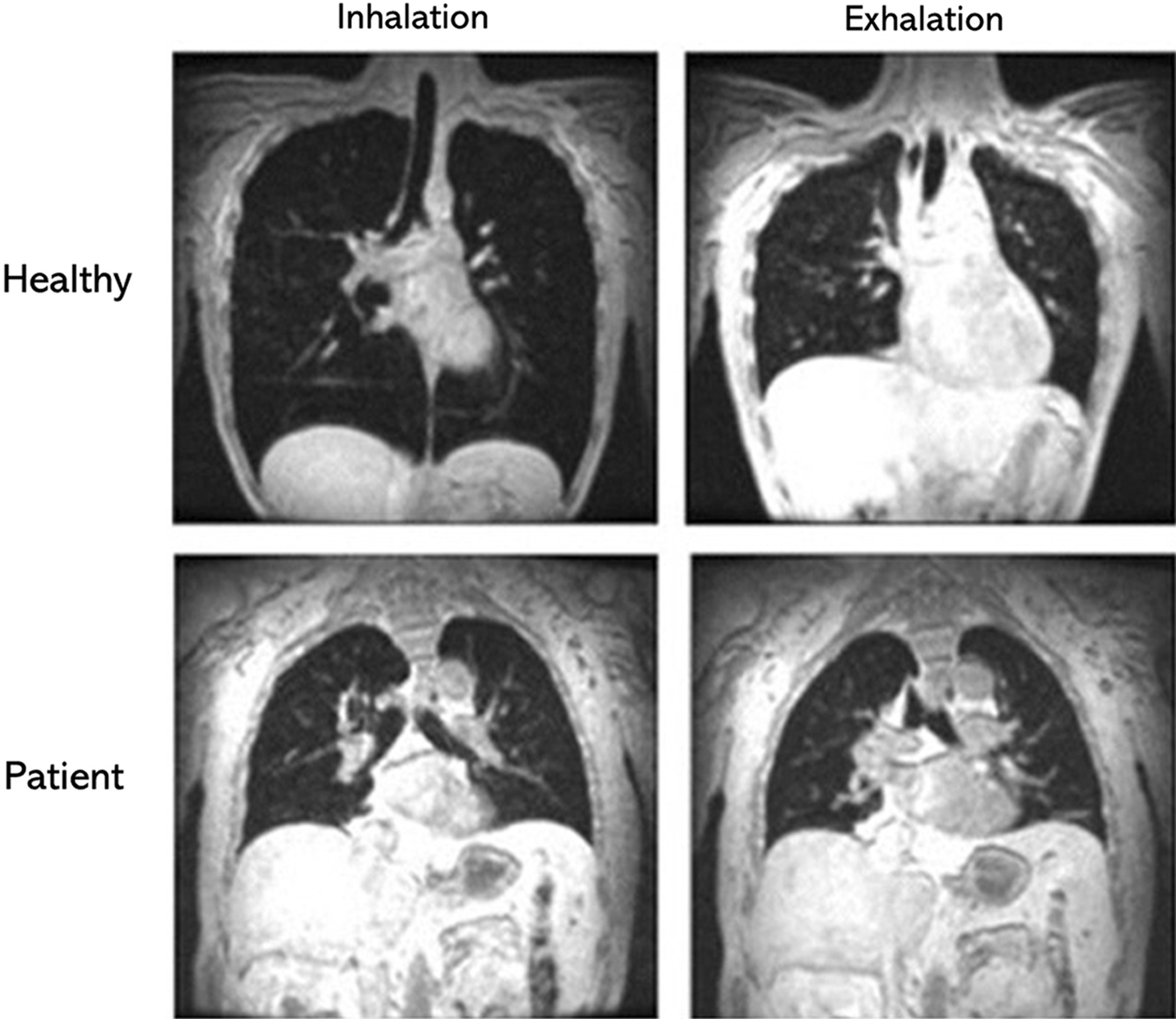
Magnetic resonance images at maximum inhalation and maximum exhalation during 12-s inspiratory breath-hold (*left panels)* and 12-s expiratory breath hold in a healthy volunteer *(upper panels)* and in a patient with Pompe disease *(lower panels)*. In the patient with Pompe disease, if there is any displacement of the diaphragm at all, is difficult to appreciate Reproduced with permission from Springer Nature: Wens et al. BMC Pulm 2015;15:54

In patients with late-onset glycogen storage disease type II, also known as late-onset POMPE disease, Gaeta et al. [54, 66] reported that diaphragm atrophy and maximal diaphragm excursions assessed with static MRI, correlate with forced vital capacity (FVC) in the supine position, standing-to-supine decrease in FVC, peak cough flow and maximal inspiratory pressure. Similar results were reported by Wens et al. [67] (Fig. 13).

In summary, chest radiograph provides limited information pertaining to diaphragm morphology [52]. The more advanced static imaging techniques, CT and MRI, provide useful information regarding the surface area and positioning of the entirety of the diaphragm within the thorax. As such, using CT and MRI, it is possible to correlate changes in diaphragm position with changes in lung volume [58, 62], and elucidate the role of the diaphragm in pulmonary disease states [54, 58]. In addition, images obtained with CT and MRI can be sufficiently detailed to detect clinically important changes in the thickness of the muscle itself; CT and MRI may become a useful tool in assessing for diaphragm atrophy [55, 56, 66].

### Non-ultrasound imaging of the diaphragm: dynamic imaging techniques

#### Fluoroscopy

Fluoroscopic imaging during a sniff maneuver is the traditional methodology used to diagnose unilateral diaphragm paralysis. During a sniff maneuver in patients with unilateral diaphragm paralysis the healthy hemidiaphragm descends, (Additional file 5) whereas the affected hemidiaphragm paradoxically ascends (Additional file 6). For the study to be considered abnormal, the affected hemidiaphragm must ascend at least 2 cm. This test has a number of limitations: it is not highly specific (6% of healthy subjects demonstrate paradoxical motion) [68] and results can be misleading in cases of incomplete paralysis or bilateral diaphragm weakness [69] (Additional file 7). Newsom-Davis et al. [69] observed paradoxical motion in less than 20% of patients with bilateral diaphragm paralysis. In patients with bilateral diaphragm weakness, the abdominal wall muscles relax at the onset of inspiration. The diaphragm descends due to the outward recoil of the abdominal wall; this movement can be misinterpreted as normal diaphragm contraction.

#### Dynamic magnetic resonance imaging

Dynamic MRI has been used to study the mechanisms responsible for the diaphragm’s shape at FRC [65], diaphragm motion in different body postures [13, 70] and to quantify the volume displaced by the contraction of the diaphragm [62].

In 1995, Gierada et al. [63] tested the feasibility of using dynamic MRI to assess diaphragm motion during slow vital capacity maneuvers. Dynamic MRI was obtained in ten healthy volunteers in the supine position. The mean excursion of the hemidiaphragm dome was 4.4 ± 0.4 (S.E.) cm on the right and 4.2 ± 0.3 cm on the left. Diaphragm displacement revealed a gradient of excursion that increased from anterior, to middle, to posterior (p < 0.05). Excursion of the lateral aspect of both hemidiaphragms was greater than that of the corresponding medial aspect (p < 0.001). Takazakura et al. [13], expanded on these findings by recording dynamic MRI in ten healthy men while sitting and while supine. The investigators reported that the movement of the diaphragm during a slow vital capacity (VC) maneuver in the supine position was greater than in the sitting position, most notably, posteriorly. The mean craniocaudal excursion of the posterior portion of the right hemidiaphragm was 10.25 ± 1.96 (S.E.) cm in the supine position and 7.95 ± 2.25 cm in the sitting position. The corresponding values for the left hemidiaphragm were 9.23 ± 2.05 cm and 8.04 ± 2.41 cm, respectively.

The coordinated contraction of diaphragm and abdominal muscles increases intra-abdominal pressure. This increase stiffens the lumbar spine and contributes to spinal stabilization during trunk and voluntary limb movements [71]. Dynamic MRI has been used to examine this stabilizing function of the diaphragm during postural limb activities [71]. In thirty healthy subjects, Kolar et al. [71] obtained dynamic MRI during tidal breathing while subjects relaxed all four extremities along the torso, and while maintaining isometric flexion of the upper or of the lower extremities against external resistance. Tidal excursions of the diaphragm were greater during upper and lower extremity contraction than during relaxed condition (p < 0.05). In addition, the position of the diaphragm at end inhalation during upper or lower extremity contraction was lower (more caudal) than during relaxed conditions (p < 0.01). The position of the diaphragm at end exhalation was lower (more caudal) during lower extremity contraction than during upper extremity contraction or during relaxed conditions (p < 0.01). The latter finding suggests that during lower extremity contraction, the diaphragm does not relax fully and remains in higher tonic state of activity. In turn, the higher tonic state of activity supports critical involvement of the diaphragm in stabilizing the spine during postural activity.

Dynamic MRI has been used for the evaluation of diaphragm movements in patients with COPD. Unal et al. [72], reported that during slow VC maneuvers, the cephalocaudal excursion of the dome of the diaphragm in 26 patients with COPD was less than half that recorded in 15 healthy subjects. Later, these same investigators reported an increase in diaphragm excursion following administration of theophylline in 26 out of 30 patients with COPD [73]. The mean increase in diaphragm excursion was 8.73 ± 1.17 (S.D.) mm. Lack of blinding and a lack of a control group limit the internal validity of this study.

In summary, of the above dynamic imaging techniques, fluoroscopy is the traditional (and more widely available) technique used in the evaluation of suspected unilateral diaphragm weakness, but it is not a specific test and results of the test can often confound the diagnostic process [69]. Dynamic MRI is not widely available, but this technique has great potential in that it can image, in real time, the coordinated movement of the lungs, muscles and adjacent structures throughout the respiratory cycle [71] and help elucidate the role of the diaphragm in pulmonary disease states [72].

### Clinical applications

Diaphragm dysfunction can present as weakness, paralysis and eventration [8, 74]; imaging modalities can aid in the assessment of these clinical conditions.

#### Ultrasound imaging in the identification of diaphragm weakness and paralysis

Diaphragm ultrasound of patients with diaphragm weakness and paralysis can demonstrate abnormalities of thickness, thickening and motion.

##### Ultrasound: diaphragm thickness

Ultrasound measurements of diaphragm thickness have been used to identify diaphragm atrophy in patients with neuromuscular disorders [32, 75] and in mechanically ventilated patients [21, 76–78].

**Fig. 14:**
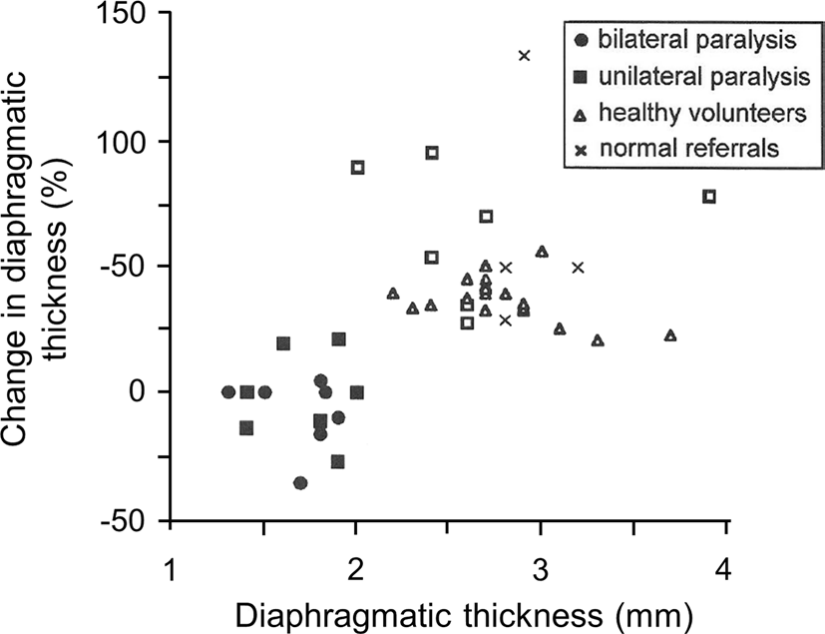
Values of diaphragm thickness at resting functional residual capacity (FRC) and percentage increase in thickness on inhaling from FRC to total lung capacity (TLC) in 5 patients with bilateral diaphragm paralysis, in 7 patients with unilateral diaphragm paralysis, 3 patients with normal function, and 15 healthy volunteers. A paralyzed diaphragm *(solid symbols)* could be distinguished from a functioning diaphragm *(open symbols)* by a resting thickness of less than 2 mm and a less than 20% increase in thickening on inhaling to TLC. Reproduced with permission from The American Thoracic Society: Gottesman et al. Am J Respir Crit Care Med 1997;155(5):1570–4

In seven patients with unilateral diaphragm paralysis, Gottesman and McCool [32] found that the paralyzed hemidiaphragm was thinner than the healthy hemidiaphragm, l.7 ± 0.2 and 2.7 ± 0.5 (S.D.) mm, respectively. A resting thickness at FRC of less than 2 mm combined with less than 20% increase in thickness on inhalation from FRC to TLC, discriminated between a paralyzed and normal diaphragm (Fig. 14). Of note, some healthy subjects may have a thickness ratio close to 20%; in such individuals, the diagnosis of paralysis would remain uncertain [44].

Noda et al. [75] assessed diaphragm thickness in 37 patients with a variety of neuromuscular disorders. Compared with diaphragm thickness of 1.68 (range 1.29–2.40) mm in 10 healthy subjects, diaphragm thickness in 16 patients with ALS was 1.08 (range 0.67–1.61) mm, in 11 patients with myopathies it was 1.12 (0.55–1.54) mm, and in 10 patients with neuropathies it was 1.41 (0.98–2.03) mm. Twenty-three patients (62.2%) had values below the lowest value in the control group. It is not clear if the investigators standardized the intercostal space where the thickness of the diaphragm was measured.

Grosu et al. [76] were the first to prospectively measure diaphragm thickness using ultrasound imaging during invasive ventilation. Diaphragm thickness decreased at an average rate of 6% per day suggesting development and a rapid progression of diaphragm atrophy. These results [76] have been extended by Zambon et al. [24] who measured diaphragm thickness daily, from the first day of mechanical ventilation until ICU discharge in 40 patients on various levels of ventilator support. The mean daily decrease in diaphragm thickness seemingly was more pronounced with increasing levels of ventilatory support. These results must be interpreted with great caution as the level of ventilator support was only marginally correlated with daily rate of diaphragm atrophy (r^2^ = 0.14, p = 0.006).

**Fig. 15:**
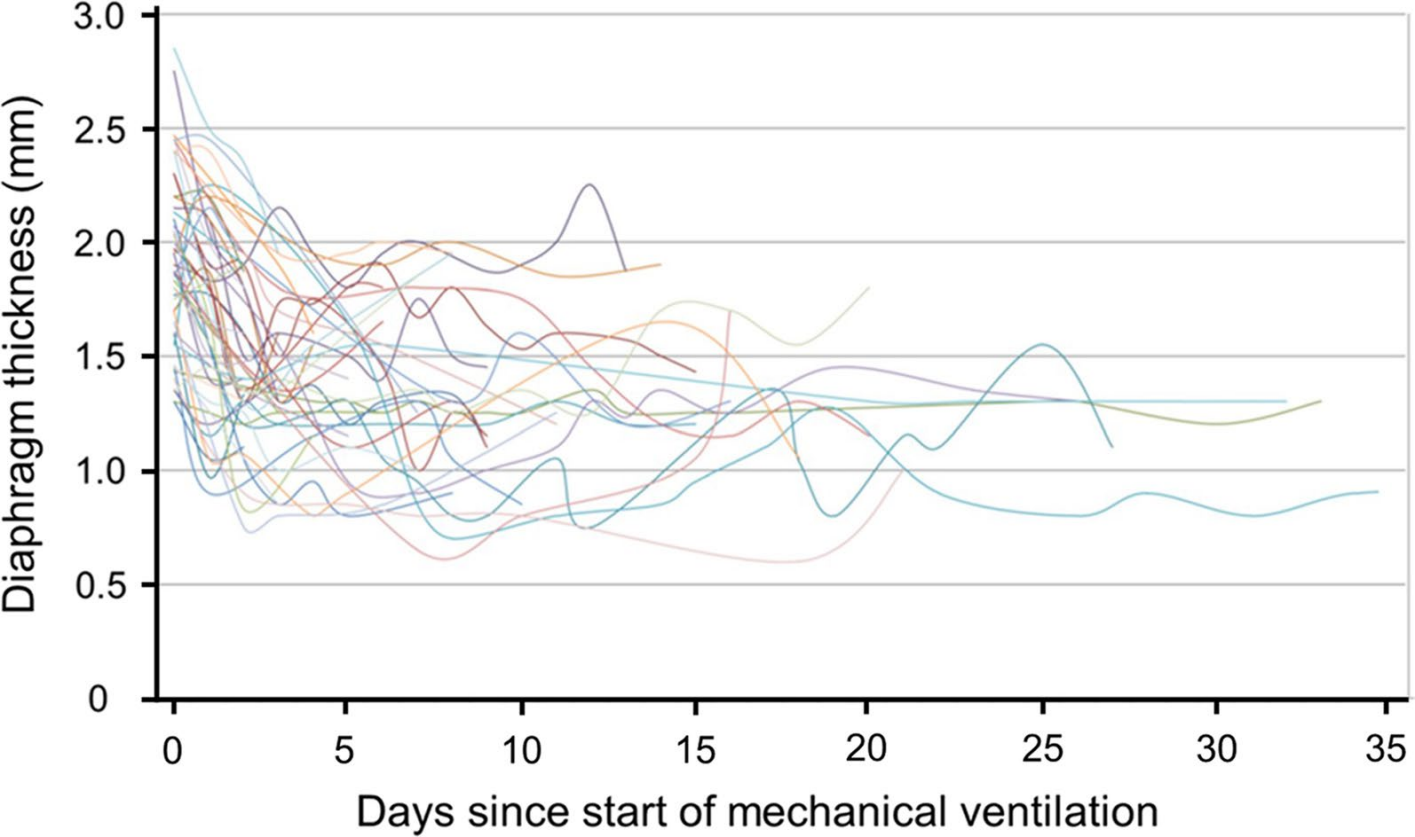
Ultrasound measurements of diaphragm thickness in critically ill patients requiring mechanical ventilation. Raw values of diaphragm thickness for each day of mechanical ventilation. Lines use a Bézier curve construction (see text for details). Reproduced under Open Access Creative Commons License: Schepens et al. Crit Care 2015;19(1):422

When examining the daily change in diaphragm thickness of individual patients in the study of Zambon et al. [24], it is apparent that some patients experienced a decrease in diaphragm thickness while others experienced no change or even an increase in thickness. Similar results have been reported by Schepens et al. [77] in 54 ventilated patients (Fig. 15). Compared to baseline, the last recorded value of thickness before extubation, tracheotomy or death had decreased in 40 (77%) patients, it was unchanged in ten (19%) patients and it had increased in two (4%) patients. It is unknown whether the latter finding is a surrogate marker of load-induced muscle swelling [79] or of ineffective compensatory myofiber hypertrophy [80, 81].

Variability in the change of diaphragm thickness was reported also in a study of more than 200 ventilated patients cared for in three Canadian ICUs [21]. Forty percent of patients developed a decrease in diaphragm thickness by day 4 of ventilation. This decrease was associated with a lower probability of weaning success and increased risk of complications (reintubation, tracheostomy, prolonged ventilation). A quarter of the patients exhibited an increase in diaphragm thickness over the first several days of ventilation. These patients were also less successful in weaning and had an increased risk of reintubation. The remaining 35% of patients experienced less than 10% change in diaphragm thickness. This last group had a greater probability of weaning success and fewer complications than the other two groups. The risk of death was similar in the three groups of patients. The investigators concluded that development of either progressive decrease or progressive increase in diaphragm thickness during the early course of mechanical ventilation increases the risk of complications and predicts prolonged ventilation. The latter conclusion, however, should be interpreted with caution as the weaning strategy and use of rescue noninvasive ventilation after extubation in the ICUs were not standardized. The study [21] also underscores a potential limitation of cross-sectional investigations of diaphragm thickness in critically ill patients. Moreover, it seriously puts into question the existence of a linear relationship between diaphragm thickness and the number (and function) of diaphragm contractile elements in these patients [82, 83]. The absence of such a relationship and, thus, the absent relationship between diaphragm thickness and clinical outcomes, is supported by the data of Dubé et al. [78], Vivier et al. [84], and Grosu et al. [85]. In the first study [78], investigators reported no correlation between PawTw elicited by magnetic stimulation of the phrenic nerves and diaphragm thickness in 99 intubated patients recovering from respiratory failure. In the second study [84], investigators reported no association between diaphragm atrophy and clinical outcomes in 35 patients receiving mechanical ventilation. In the third study [85], investigators reported that among 57 patients receiving mechanical ventilation, paradoxically, the thinner the diaphragm at baseline and the greater the extent of diaphragm thinning at 72 h of mechanical ventilation, the greater the likelihood of successful extubation.

##### Ultrasound: diaphragm thickening

Measurements diaphragm thickening as a surrogate measurement of the intensity of voluntary diaphragm contraction has been used in patients with phrenic neuropathy and in critically ill patients. Summerhill et al. [86] measured diaphragm thickening in 16 patients with phrenic neuropathy and followed them for up to 60 months, and concluded that ultrasound may be useful for assessing functional recovery from diaphragm weakness.

**Fig. 16:**
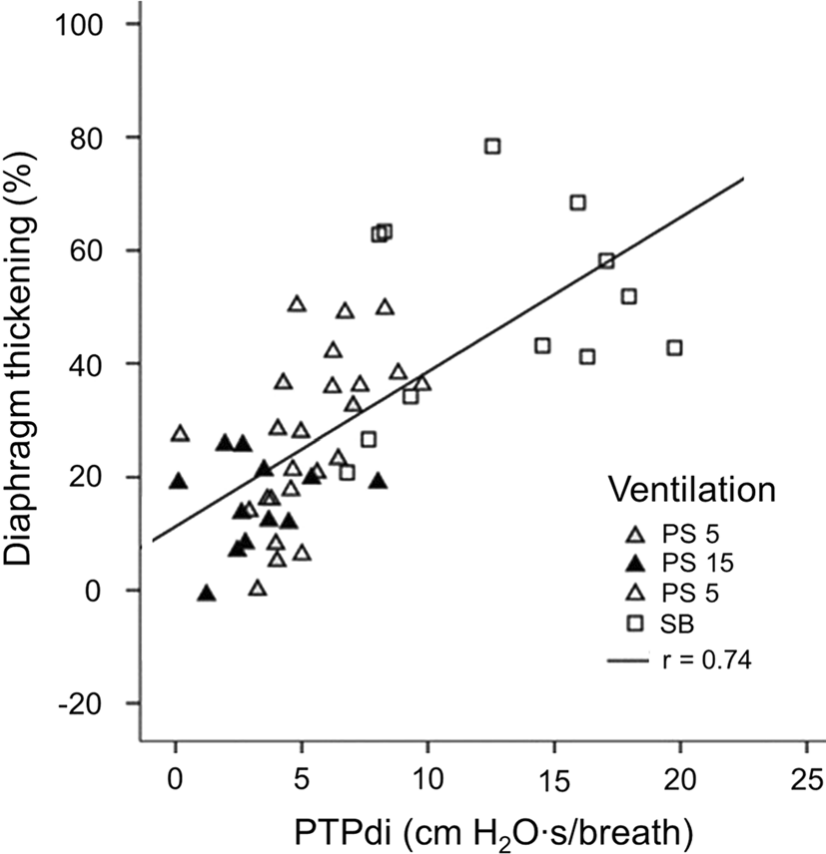
Relationship between the thickening of the diaphragm recorded with ultrasonography and changes in the pressure–time product of the diaphragm (PTPdi) in 12 patients during pressure support ventilation set at 5, 10, 15 cm H_2_O (PS 5, PS 10 and PS 15, respectively) and during unsupported breathing (SB). Diaphragm thickening increased as PTPdi increased. The correlation between diaphragm thickening and PTPdi was significant (ρ = 0.74, p < 0.001) yet, the interindividual variability was high. Reproduced with permission from Springer Nature: Vivier et al. Intensive Care Med 2012;38(5):796–803

Diaphragm thickening has been used to assess diaphragm dysfunction in critically ill patients during non-invasive [34] and invasive ventilation [21, 28, 51, 87]. Vivier et al. [34] compared diaphragm thickening and the pressure output of the diaphragm in terms of pressure time product of the diaphragm (PTPdi) in 12 patients requiring non-invasive ventilation after extubation (Fig. 16). Although the correlation between PTPdi and diaphragm thickening was significant (ρ = 0.74, p < 0.001), the interindividual variability was high. For example, diaphragm thickening associated with a PTPdi of about 9 cm H_2_O·s/breath ranged from less than 20% to more than 60%. Similar results have been reported by Umbrello et al. [51] in 25 intubated patients.

Dubé et al. [78], compared diaphragm thickening and PawTw in 99 intubated patients. The correlation between PawTw and diaphragm thickening was significant (p = 0.87, p < 0.001) yet, as with Vivier et al. [34], the interindividual variability was high. For example, the diaphragm thickening associated with a PawTw of about 9 cm H_2_O ranged from about 20% to about 40%. By multiple linear regression model analysis, diaphragm thickening less than 29% was an independent predictor of duration of mechanical ventilation and ICU and hospital mortality. None of these results have been prospectively validated.

Canadian investigators [21] have suggested a causal link between ventilator-associated decrease in diaphragm thickness and abnormally low inspiratory effort (reduced diaphragm thickening) and between ventilator-associated increased thickness and excessive effort (increased diaphragm thickening). These speculations are challenged by several considerations. The correlation between thickening and effort is tenuous: ultrasound measurements of diaphragm thickening explain one third (or less) of the variability in inspiratory effort (Fig. 4) [28, 34, 51]—or even none at all [36]. Measurements of diaphragm thickening (and diaphragm excursions) obtained with ultrasonography are marred by limitations such as angle dependence and translational error (where other areas of diaphragm move into the ‘line of sight’) [25]. Not surprisingly, investigators have reported that diaphragm thickening is frequently decreased after elective cardiac surgery without this having an impact on weaning outcome [88]. The extent of diaphragm thickening for a given level of inspiratory effort varies considerably between subjects [37, 51, 87]. The within-session reproducibility of ultrasound measurements of diaphragm thickening is weak [28]. Finally, in the Canadian study [21], ultrasound recordings of diaphragm thickening where obtained over a limited period of time once a day, five days a week. This limits the generalizability of the study considering that during mechanical ventilation patient-ventilator interaction is not uniform over time [89].

**Fig. 17:**
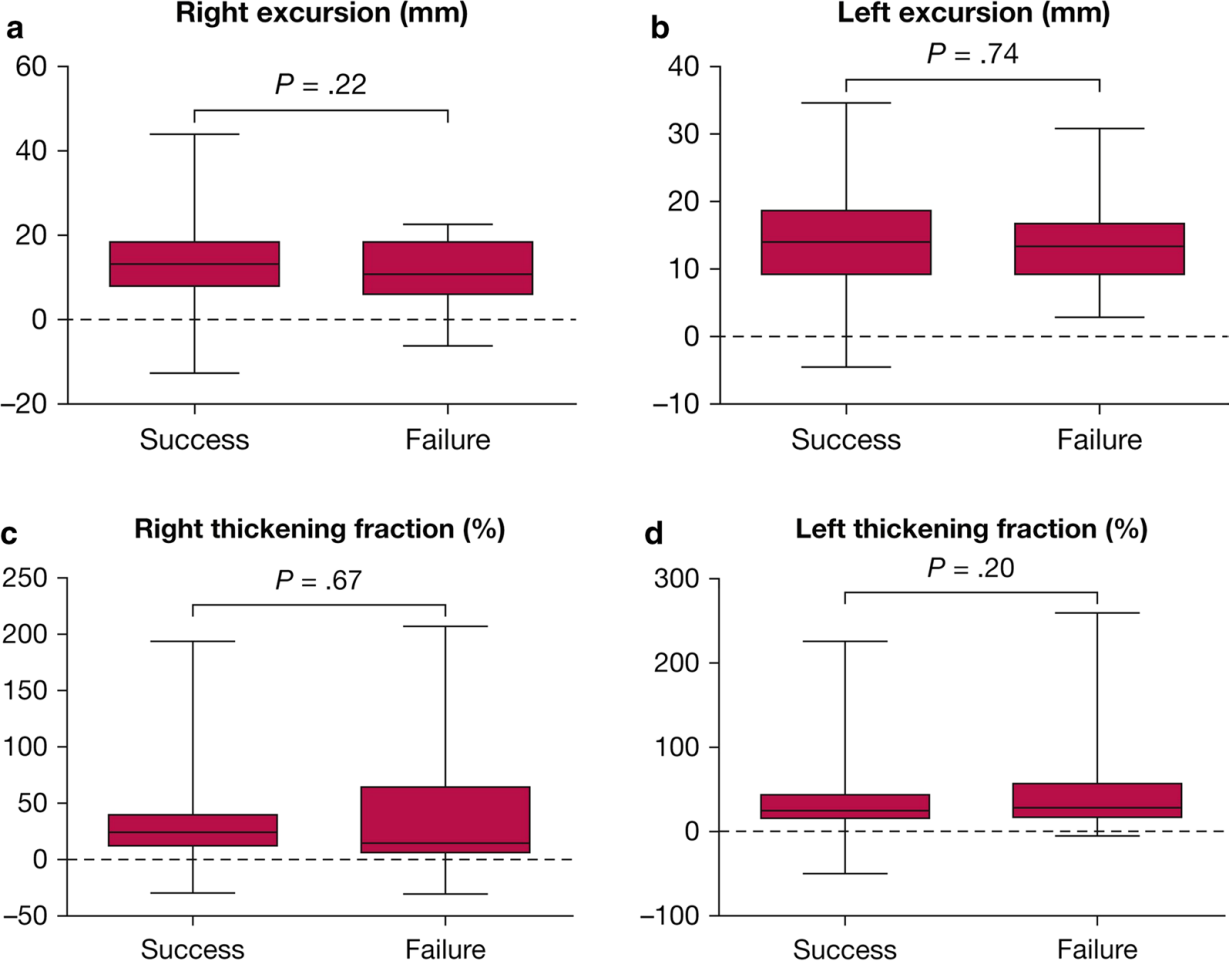
Right and left hemidiaphragm excursion and thickening in more than 150 patients who succeeded or failed extubation from mechanical ventilation. Excursion *(upper panels)* of the right (**a**) and left (**b**) hemidiaphragms. There was no difference in hemidiaphragm excursions between patients whose extubation succeeded and those whose extubation failed. Negative values reflect paradoxical movements (passive ascent) associated with complete hemidiaphragm paralysis. Thickening fraction (*lower panels*) of the right **(c)** and left (**d**) hemidiaphragms. There was no difference in hemidiaphragm diaphragm thickening between patients whose extubation succeeded and those whose extubation failed. Negative values reflect paradoxical movements (passive thinning) associated with complete hemidiaphragm paralysis. Hemidiaphragm excursions and thickening are presented by a box plot representation with median, 25th and 75th percentiles, and adjacent values. Reproduced with permission from Elsevier: Vivier et al. Chest 2019;155(6):1131–9

DiNino et al. [90] prospectively assessed the value of diaphragm thickening during tidal breathing to predict extubation success in 63 ventilated patients. The combined sensitivity and specificity of a diaphragm thickening value ≥ 30% for extubation success was 88% and 71%, respectively. The area under the receiver operating characteristic curve was 0.79. Similar results have been obtained by Ferrari et al. [87] (diaphragm thickening > 36% was associated with extubation success) and Pirompanich and Romsaiyut [91] (diaphragm thickening > 26% was associated with extubation success). None of these thresholds has been prospectively validated. In a recent multicenter trial, the proportion of patients with diaphragm dysfunction was similar between extubation successes and extubation failures: 71% vs. 68% (p = 0.73) (Fig. 17) [92]*.* The investigators concluded that diaphragm dysfunction assessed by ultrasound is not associated with an increased risk of extubation failure.

##### Ultrasound: diaphragm excursions

Ultrasound measurements of diaphragm excursions have been used to assess diaphragm function in patients with a variety of neuromuscular disorders [44, 46, 93, 94], after surgery [47, 95], and in critically ill patients requiring mechanical ventilation [48, 51, 78].

In 45 patients, most of whom had amyotrophic lateral sclerosis or myotonic dystrophy, Carrié et al. [94] reported that the relationship between excursions of the right hemidiaphragm during deep breathing and percent predicted FVC was weak: r^2^ = 0.52 (p < 0.0001). Mechanisms responsible for these disappointing results include abnormal respiratory mechanics, variable recruitment of the hemidiaphragm and of the rib cage and accessory respiratory muscles.

**Fig. 18:**
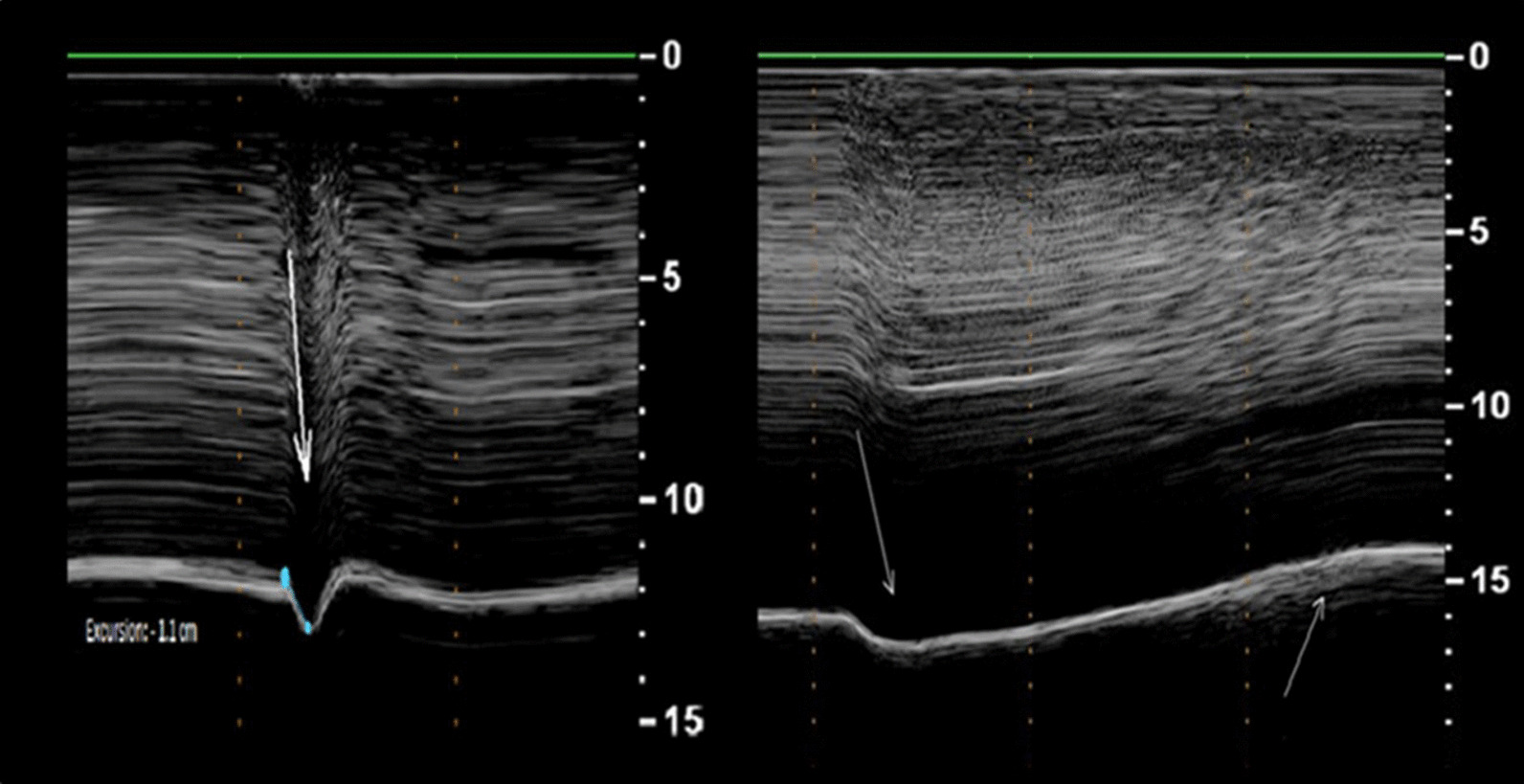
M-mode ultrasonography in a representative patient with hemidiaphragm paralysis recorded during a sniff (left panel) and during deep breathing (right panel). During a sniff (left panel), the paralyzed hemidiaphragm show a paradoxical motion (arrow). During deep breathing (right panel), the hemidiaphragm displays a biphasic movement characterized by an initial paradoxical movement (left arrow) and a terminal caudal displacement (right arrow). (Reproduced with permission from John Wiley and Sons: Boussuges et al. Clin Physiol Funct Imaging 2019;39(2):143–9)

Boussuges et al. [44] recorded diaphragm motion in 26 patients with known hemidiaphragm paralysis. During quiet breathing the paralyzed hemidiaphragm had either no detectable motion or a weak paradoxical (cranial) displacement (less than 0.5 cm). During sniffing and deep breathing, a paradoxical motion of the paralyzed hemidiaphragm of approximately 10 mm or more was recorded in all patients (Fig. 18). Excursions of the non-paralyzed hemidiaphragm, during quiet breathing and voluntary sniffing, but not during deep breathing, were greater in patients than in healthy controls. Despite the encouraging results, a major limitation of the study acknowledged by the investigators, is that the study was not designed to detect diaphragm paralysis but to describe the ultrasonographic characteristics in patients with known diaphragm paralysis.

**Fig. 19:**
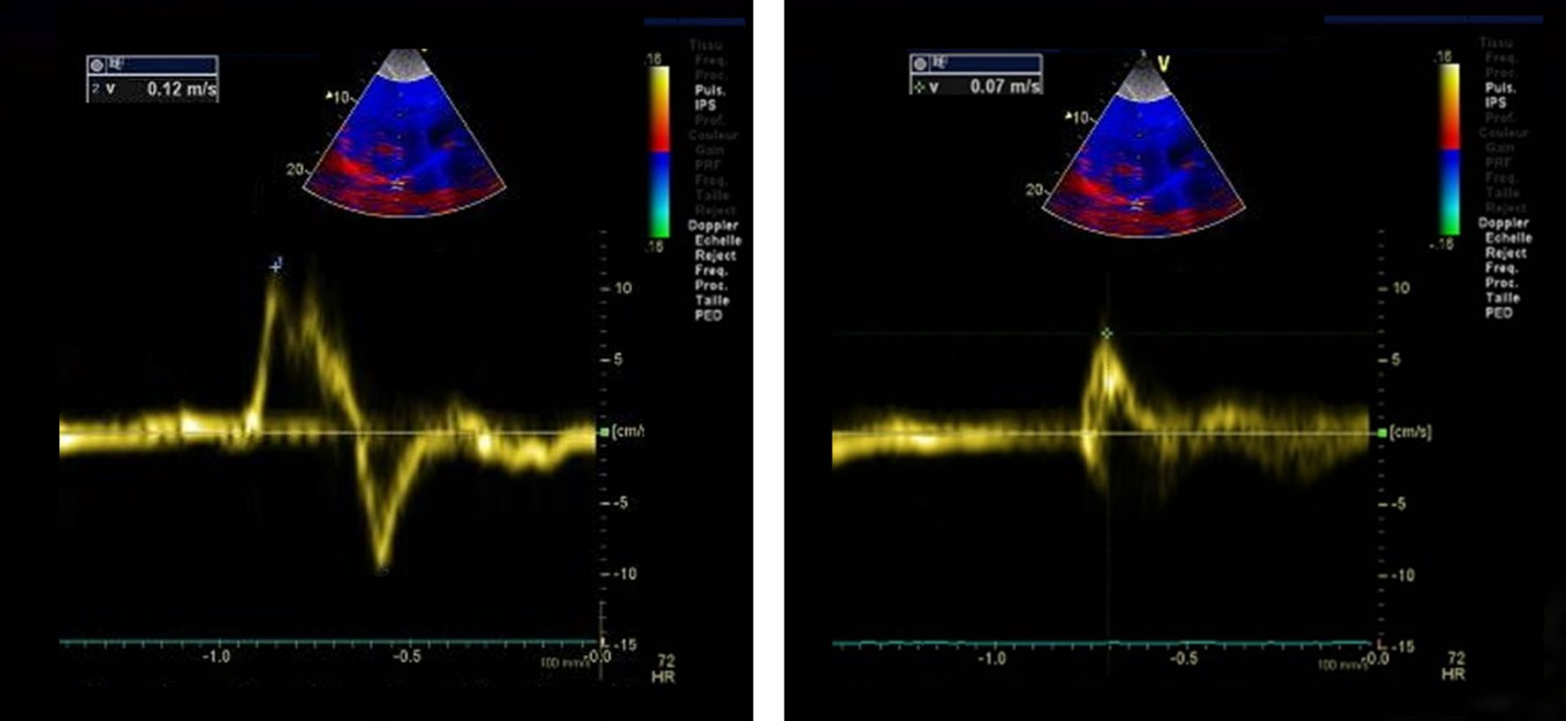
Representative tracings of peak velocity of the right hemidiaphragm during a sniff maneuver using tissue Doppler imaging in a healthy volunteer (*left panel*) and in a patient with Duchenne muscular dystrophy (*right panel).* The peak velocity in the healthy subject (12 cm/s) was nearly as twice as fast as the peak velocity in the patient Duchenne muscular dystrophy (7 cm/s). Reproduced under Open Access Creative Commons License: Fayssoil et al. PLoS One 2019;14(4):e0214288

Fayssoil et al. [96] built upon the results of Boussuges et al. [44] by recording diaphragm sniff ultrasound in 89 patients with a variety of neuromuscular disorders. Sniff diaphragm motion was associated with sniff nasal pressure with a correlation coefficient (r) ranging from 0.60 to 0.63 that would correspond to a coefficient of determination (r^2^) of only about 0.30—i.e., sniff diaphragm motion would explain about 30% of the variance in sniff nasal pressure. Fayssoil et al. [96] measured the peak sniff inspiratory velocity (cm/s) using diaphragm tissue Doppler imaging (Fig. 19); correlation coefficient between sniff nasal pressure and peak sniff inspiratory velocity remained poor. The investigators recognize the technical difficulties in using diaphragm tissue Doppler imaging.

In intubated patients, diaphragm excursions are not related to the muscle’s pressure output recorded during voluntary contractions (PTPdi) [51], and only marginally related to the muscle’s pressure output elicited by phrenic nerve stimulation (PawTw) [78]. Despite these negative results, Lerolle et al. [47] assessed whether diaphragm excursions during deep breathing could be used to diagnose severe diaphragm dysfunction in 28 patients requiring mechanical ventilation for more than 7 days after cardiac surgery. Diaphragm excursions during a trial of unsupported breathing (T-tube trial), were smaller in patients with severe diaphragm dysfunction than in those without it: mean (IQR) excursion of 19 (7) mm and 30 (10) mm, respectively (p = 0.001). These results have not been prospectively validated.

##### Non-ultrasound imaging in the identification of diaphragm weakness and paralysis

**Fig. 20:**
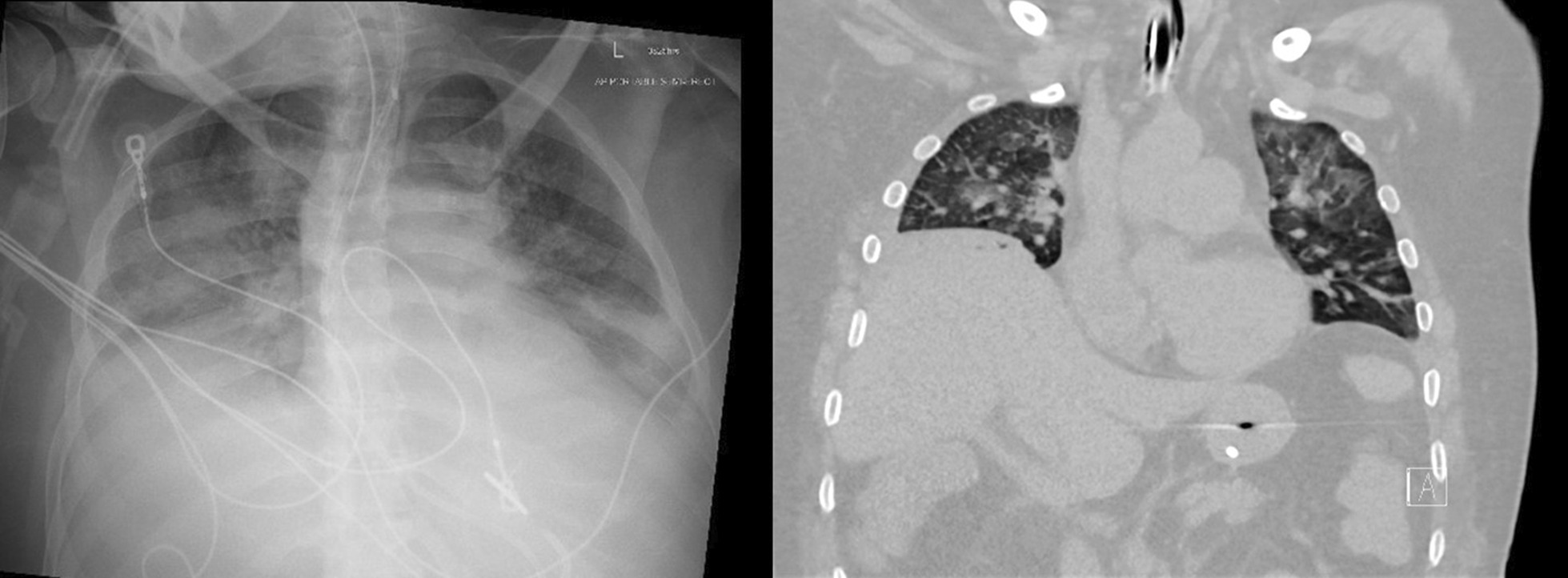
Chest radiograph (left panel) and chest CT (right panel) in a patient with myotonic dystrophy and respiratory failure. Bilateral hemidiaphragm elevation (right hemidiaphragm elevation is greater than that of the left) likely caused by diaphragm myopathy

Plain chest radiographs and CT imaging generate still images of the diaphragm and, thus, they provide information about shape and position of the muscle (Fig. 20). They cannot provide direct information on diaphragm movement and diaphragm function. Indeed, using plain chest radiographs, the correct identification of patients with unilateral diaphragm paralysis (sensitivity) is as low as 66.6% [97] and the correct identification of patients without unilateral diaphragm paralysis (specificity) is only 44% [52]. Elevation of both hemidiaphragms, along with small pulmonary volumes and bibasilar atelectasis, can be seen in patients with bilateral diaphragm paralysis. These findings, however, are not necessarily a sign of weakness or paralysis. Absence of diaphragm elevation makes diaphragm weakness or paralysis unlikely [52].

Fluoroscopy during a sniff provides two-dimensional information of the movement of (mainly) the central tendon of the diaphragm. Unfortunately, as already discussed, fluoroscopy can give misleading results particularly when assessing patients with hemidiaphragm paresis or bilateral paralysis [69]. Finally, the test is marred by the need for radiation exposure.

**Fig. 21:**
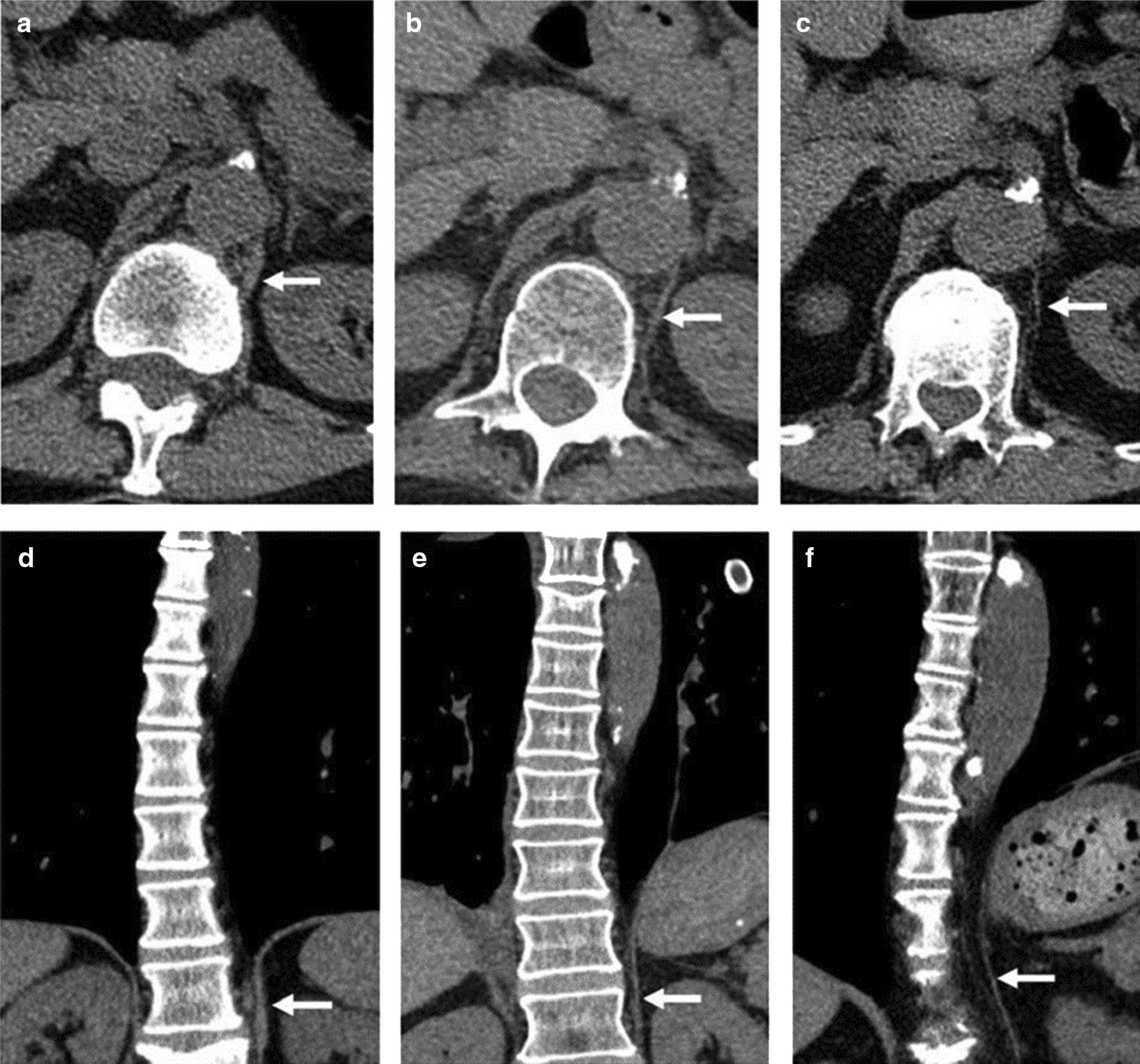
Progressive hemidiaphragm thinning after double lung transplantation for COPD. Axial (**a**–**c**) and coronal (**d**–**f**) CT images pre-transplantation (*left panels)*, 1 month after transplantation *(middle panels)* and 11 months after lung transplantation (*right panels)*. The pre-transplantation images demonstrate equal crural diaphragm thickness; the left hemidiaphragm (white arrow) becomes progressively thinner and moves cranially after transplantation. These findings are indicative of post-operative left hemidiaphragm paralysis. Reproduced with permission from Wolters Kluwer Health: Sukkasem et al. Journal of Thoracic Imaging 2017;32(6): 383–390

In one retrospective study of 72 patients with CT scans, Sukkasem et al. [56] reported that the crura of a paralyzed hemidiaphragm is, on average, 2 mm thinner than the crura of the contralateral healthy hemidiaphragm (Fig. 21). The study, however, has several limitations. The diagnosis of hemidiaphragm paralysis was based on fluoroscopy. All measurements were performed by one observer. The slice thickness of the CT was variable (range: 0.625 to 5 mm). In spite of these limitations, CT imaging of the diaphragm, remains more objective and simpler to interpret than a fluoroscopic sniff test or ultrasound studies [56].

In contrast to fluoroscopy and CT, dynamic MRI has the advantage of allowing to study of the motion of segments of the diaphragm in multiple planes [74] (Additional file 8). This technique has been used to assess diaphragmatic dysfunction and response to therapy in patients with late-onset Pompe disease, polymyositis, and myasthenia gravis and surgical plication [6, 16, 66]. Whether dynamic MRI of the diaphragm is superior to ultrasound imaging in making a diagnosis of diaphragm weakness or paralysis remains to be determined.

#### Identification of diaphragm eventration

Diaphragm eventration is an abnormal elevation of the diaphragm that retains normal attachments to the sternum, ribs and dorsolumbar spine [74]. In contrast to diaphragmatic hernias, in which there is a defect of the muscle through which the abdominal viscera may migrate into the chest, in diaphragm eventration, the continuity of diaphragm remains intact. With eventration, the diaphragm exhibits incomplete muscularization with a membranous sheet replacing normal muscle fibers. With diaphragmatic paralysis, muscle fibers—albeit atrophic—are still present [98]. Diaphragm eventration can be congenital or acquired [6]. In patients with congenital eventration, a portion of the hemidiaphragm, typically the anteromedial portion of the right hemidiaphragm, is elevated while the remaining portion is of normal height [8]. Acquired eventration is most often caused by phrenic nerve injury, and it usually involves the entirety of the hemidiaphragm (usually the left one) [6].

**Fig. 22:**
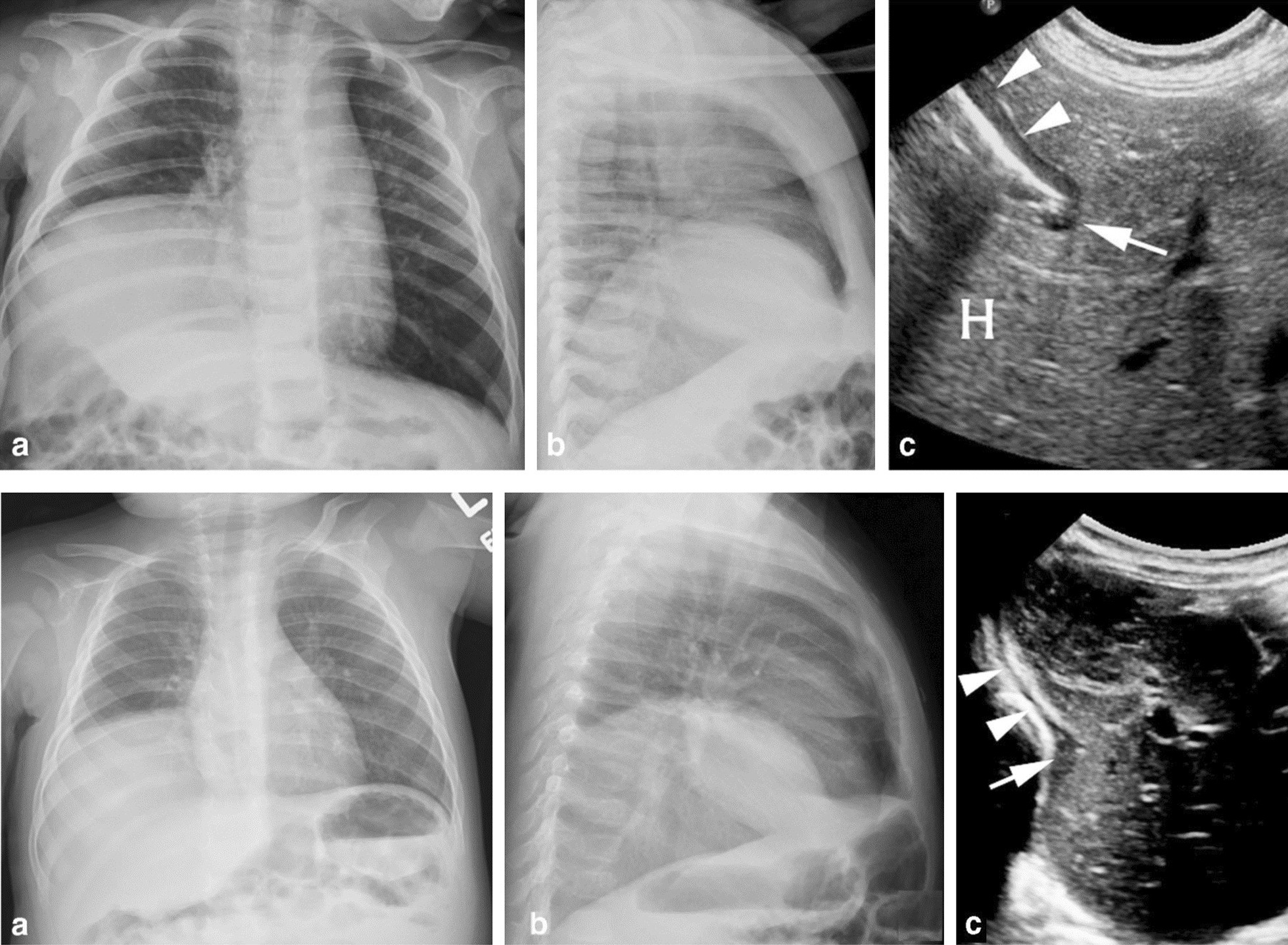
Chest radiographs and ultrasound images in representative patients with diaphragmatic hernia *(upper panels)* and diaphragmatic eventration *(lower panels). (Upper panels)* A 5-month-old boy, with right Bochdalek hernia. Anteroposterior and lateral chest radiographs demonstrate what would appear as global elevation of the right hemidiaphragm (**a**, **b**). Lateral long-view ultrasound (**c**) demonstrates the normal anterior hypoechogenic muscle (arrowheads), the folding free edge of the diaphragm at a narrow angle waist (arrow), and the herniated liver through the diaphragmatic defect (H). *(Lower panels)* A 9-month-old boy, with right diaphragmatic eventration. Anteroposterior and lateral chest radiographs demonstrate global elevation of the right hemidiaphragm (**a**, **b**). Long-view ultrasound (**c**) demonstrates posterior elevation of the diaphragm with a wide-angle waist. The normal anterior hypoechogenic diaphragm (arrowheads) with focal thickening of the muscle (arrow) covering the entire waist. Reproduced with permission from Springer Nature: Karmazyn et al. Pediatr Radiol 2019;49(8):1010–7

Karmazyn et al. [99] reported on 17 infants who had undergone diaphragm ultrasound before surgery for eventration (n = 8) or hernia (n = 9) (Fig. 22). The sensitivity and specificity of diaphragm ultrasound to diagnose hernia was 100% and 62.5%, respectively. Differentiation between eventration and hernia was not possible in about a third of infants. In adults, the use of diaphragm ultrasonography to assess for diaphragm eventration has not been validated [6, 100].

**Fig. 23:**
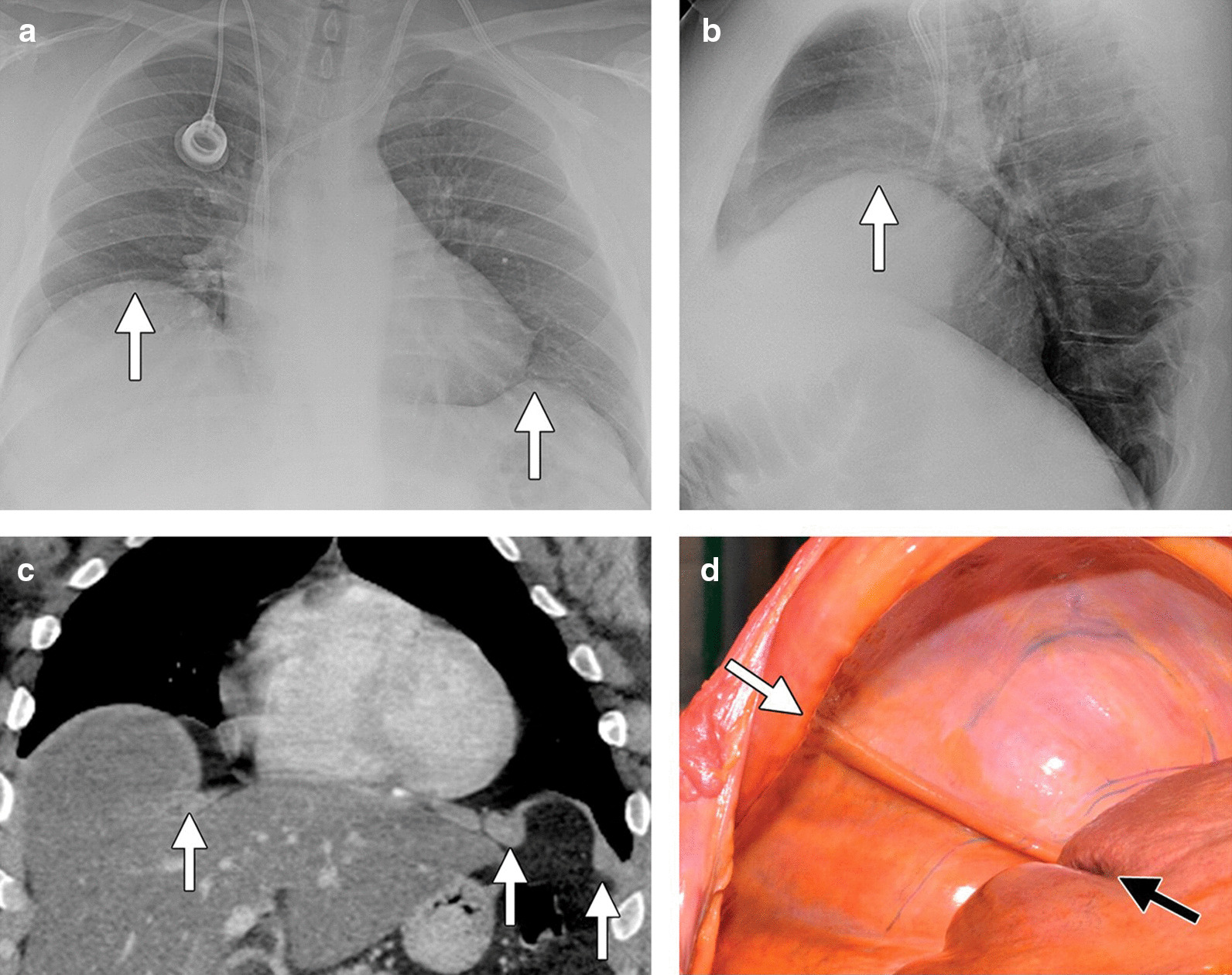
Bilateral hemidiaphragm eventration. Posteroanterior (**a**) and lateral (**b**) radiographs demonstrate focal elevation of the right and left hemidiaphragms (white arrows). Axial (**c**) CT image shows the mushroom appearance of the right and left hemidiaphragms with undercut edges (white arrows). On the right, a portion of the liver occupies the eventration herniation. Eventration (**d**) on pathology photograph showing the medial aspect (white arrow) of the muscle band and the groove on the liver created by the diaphragm (black arrow). Reproduced with permission from Radiologic Society of North America: Nason et al. Radiographics 2012;32(2):E51–E70

**Fig. 24:**
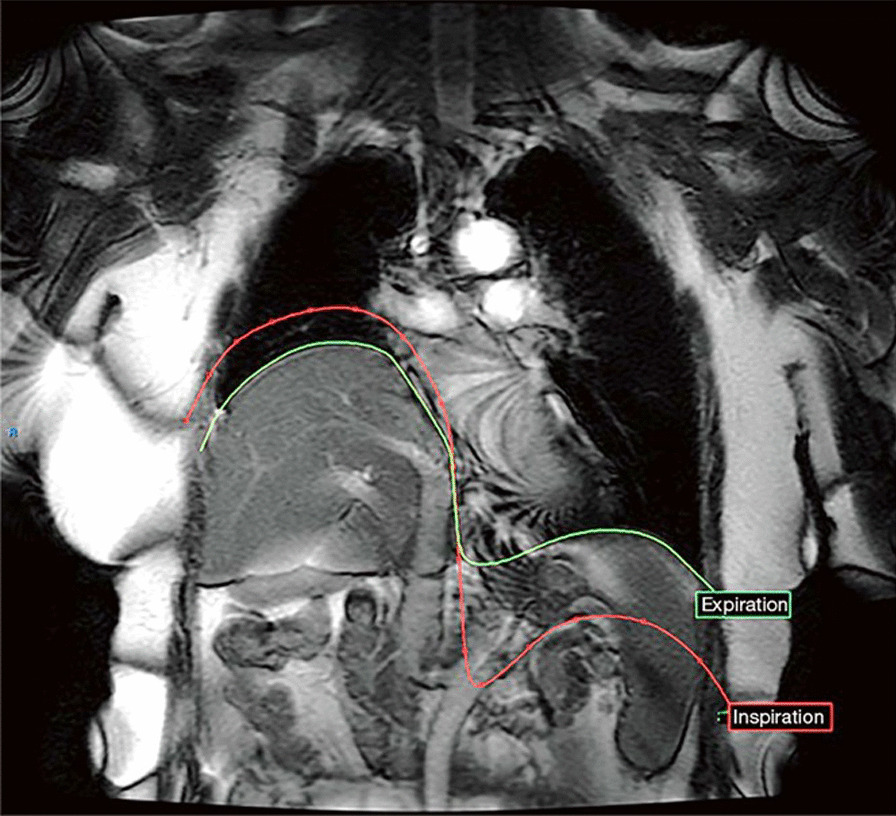
Coronal dynamic MRI of representative patient with right hemidiaphragm eventration due to hemidiaphragm paralysis. At the end of inspiration (red line), the healthy left diaphragm has descended caudally while the paralyzed right hemidiaphragm paradoxically moves cranially. At end exhalation (green line), the paralyzed right hemidiaphragm continues to be elevated relative to the left. Reproduced under Open Access Creative Commons License: Le Pimpec-Barthes et al. J Thorac Dis 2019;11(8):3467–3475

On chest radiographs, hemidiaphragm elevation can be a sign of diaphragmatic eventration, however, it is a nonspecific finding as the same may occur with diaphragm paralysis and with processes affecting the lung parenchyma (atelectasis, fibrosis), the pleural (pleural effusions, masses), and with subdiaphragmatic processes (hepatomegaly, splenomegaly, gastric dilatation, and subphrenic abscesses). The role of fluoroscopy in establishing a diagnosis of diaphragm eventration remains controversial [74] (Additional file 9). On CT, the affected portion of the diaphragm can have undercut edges or a ‘mushroom’ appearance (Fig. 23). MRI has also been used to characterize the extent of diaphragm eventration [6] (Fig. 24).

#### Etiology and prognosis of diaphragm dysfunction

Respiratory muscle dysfunction may be a clinical concern in critically ill patients who are difficult to wean from mechanical ventilation [101–103]. Diaphragm ultrasound is an increasingly available, noninvasive imaging modality familiar to most intensivists. Diaphragm ultrasound measurements, however, must be interpreted with extreme caution [104]. Use of diaphragm ultrasound to predict weaning outcome has yielded inconsistent results [35, 87, 91, 92, 105]. Most mechanically ventilated patients diagnosed with diaphragm dysfunction are successfully extubated [106–108]. To date, diaphragm ultrasound parameters cannot supplant standard weaning indices or clinical judgement [104].

**Fig. 25:**
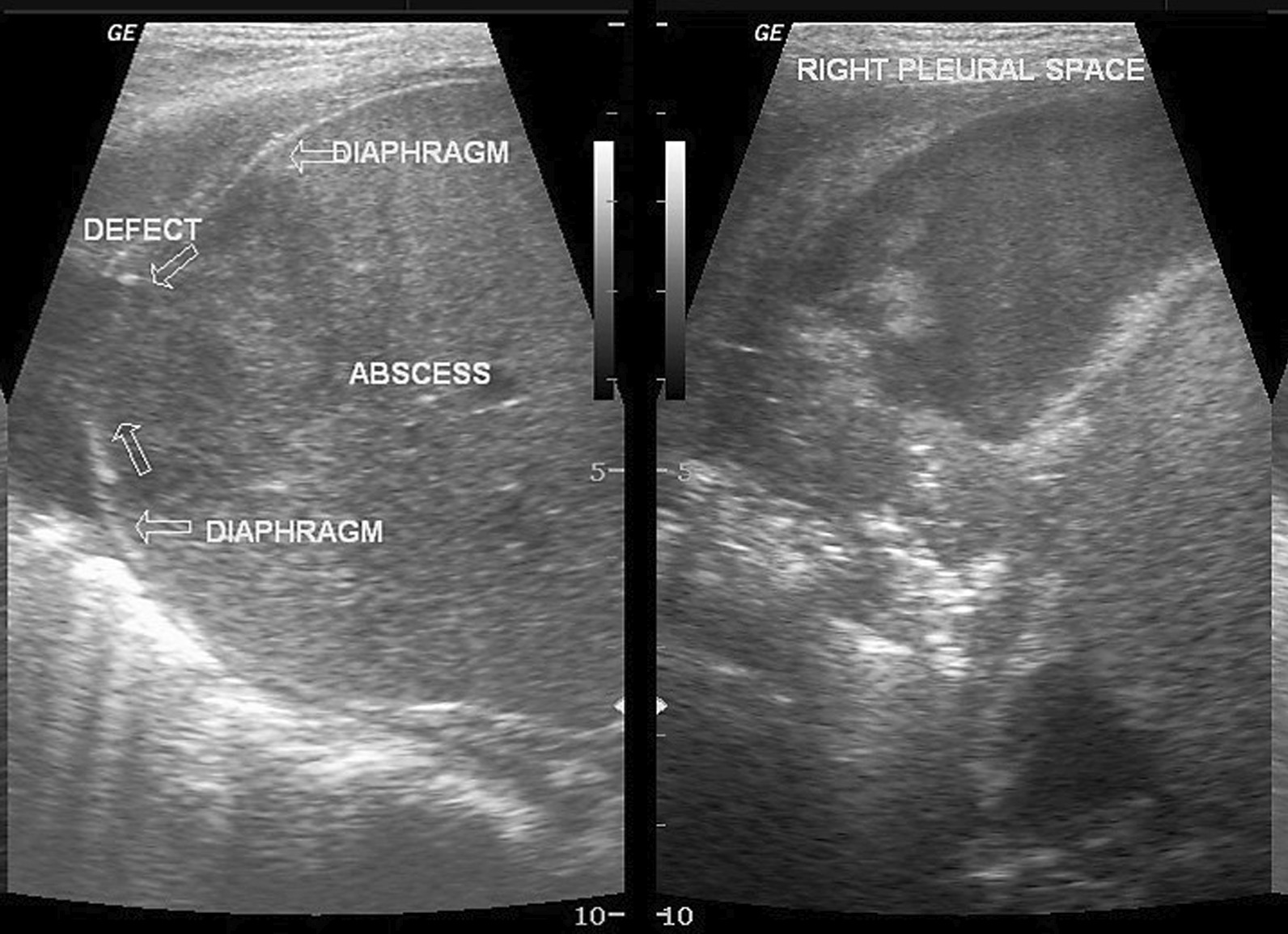
Hepatic abscess in a 3-year-old boy who presented with fever and abdominal pain. The rupture of the hepatic abscess led to a defect of the right hemidiaphragm (*left panel*) and development of right-sided empyema (*right panel*). Case coutesy of Dr Maulik S Patel, Radiopaedia.org, rID: 12,772

Ultrasound imaging can assist in identifying pathological processes extrinsic to the diaphragm such as pleural fluid, subphrenic and hepatic abscesses, and thoracic masses [15, 109], and processes intrinsic to the diaphragm such as eventration, hernias and rupture (Fig. 25). Unfortunately, the specificity of the “focused assessment with sonography for trauma” (FAST) to diagnose diaphragm rupture is only 50% [110].

**Fig. 26:**
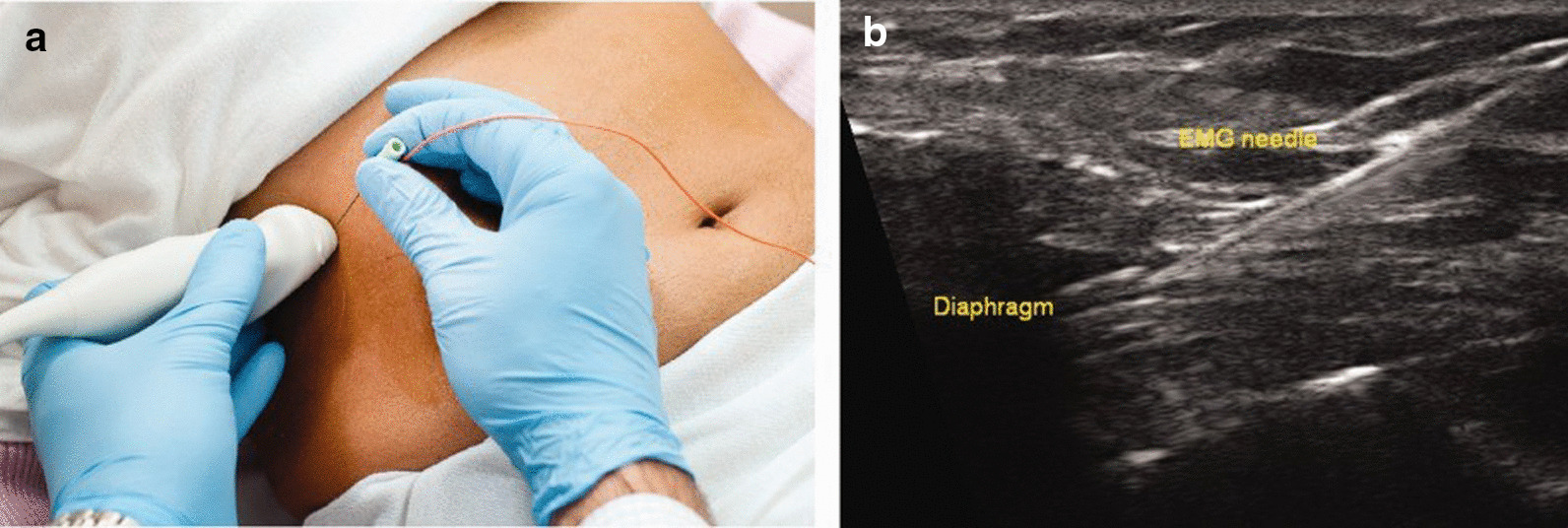
Ultrasound guidance for needle electromyography. (*Left panel, ***a**) Setup for real‐time ultrasound imaging of the diaphragm during placement of a needle electrode. (*Right panel, ***b**) Needle electrode penetrating the zone of apposition of the diaphragm. Reproduced with permission from John Wiley and Sons: Sarwal et al. Muscle Nerve 2013;47(3):319–29

Occasionally, patients suspected of having diaphragm dysfunction require electromyographic examination of the diaphragm to distinguish between upper and lower motor neuron disease using needle electrodes [1, 15]. Ultrasound imaging of the diaphragm while inserting the needle electrodes can improve safety and diagnostic value of this invasive procedure (Fig. 26).

**Fig. 27:**
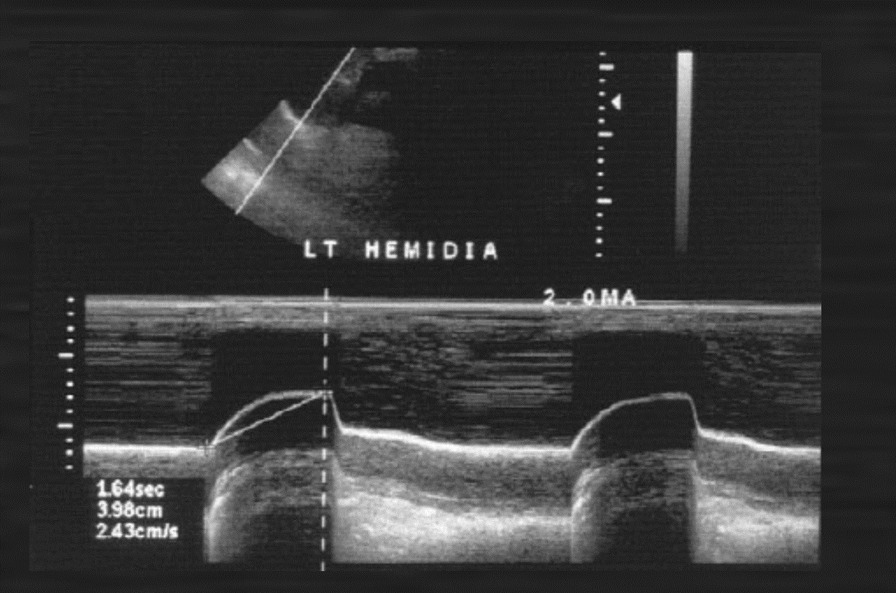
Paralyzed left hemidiaphragm with implanted electronic pacer. M-mode imaging shows diaphragm motion in response to the pacer triggering. Reproduced under Open Access Creative Commons License: Gerscovich et al. J Ultrasound Med 2001;20(6):597–604

Ultrasonography has been used also to assess the severity of diaphragm dysfunction in patients with neurological disorders such as neuralgic amyotrophy [86], ALS [75], cerebrovascular accidents [111], and also to adjust or monitor diaphragmatic pacers (Fig. 27) [15, 112] and to assess the severity of diaphragm dysfunction after abdominal [113] or thoracic surgery [114].

Patients with Duchenne muscular dystrophy exhibited greater diaphragm thickness at FRC than age-matched controls, 1.74 ± 0.21 and 1.48 ± 0.20 (S.D.) mm, respectively [115], this despite considerable impairment of maximal inspiratory mouth pressure (Pimax) (-37 ± 8 cm H_2_O, controls -80 ± 33 cm H_2_O). This finding could be analogous to the pseudo-hypertrophy observed in some limb muscle groups in these patients [115]. Summerhill et al. [86] studied 16 patients with unilateral (n = 10) and bilateral (n = 6) diaphragm paralysis and measured diaphragm thickening for up to 60 months. Eleven of 16 patients recovered from diaphragm paralysis (mean recovery time was 14.9 ± 6.1 (S.D.) months). The investigators also reported a positive correlation between improvement in diaphragm thickening and interval changes in VC, Pimax, and end-expiratory diaphragm thickness. These results suggest that ultrasonography may be used to assess functional recovery from diaphragm weakness and diaphragm paralysis.

#### Pathologic lesions and diaphragm dysfunction

**Fig. 28:**
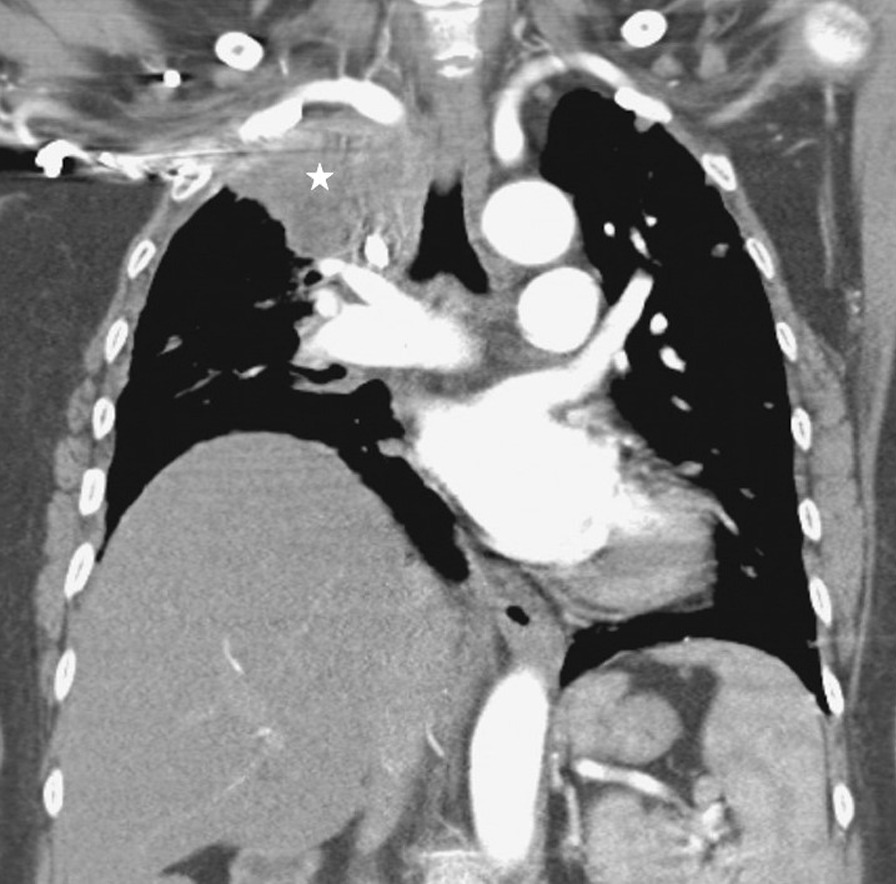
Right hemidiaphragm paralysis due to phrenic nerve invasion by apical pulmonary metastasis from colorectal carcinoma. Coronal CT image shows right apical mass (star) and right hemidiaphragm elevation. Case coutesy of Assoc Prof Frank Gaillard, Radiopaedia.org, rID: 8543

**Fig. 29:**
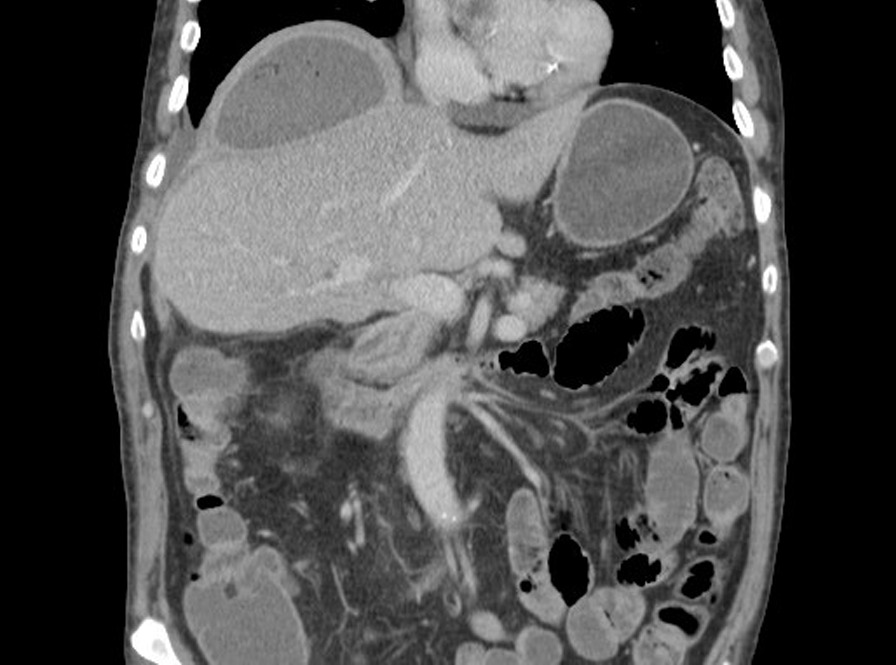
Coronal CT image in a patient with fever and abdominal pain one week after cholecystectomy. There is elevation of the right hemidiaphragm with an encapsulated collection containing fluid and gas between the diaphragm and the surface of the liver consistent with subphrenic abscess. Case coutesy of Dr Vitalii Rogalskyi, Radiopaedia.org, rID: 66300

**Fig. 30:**
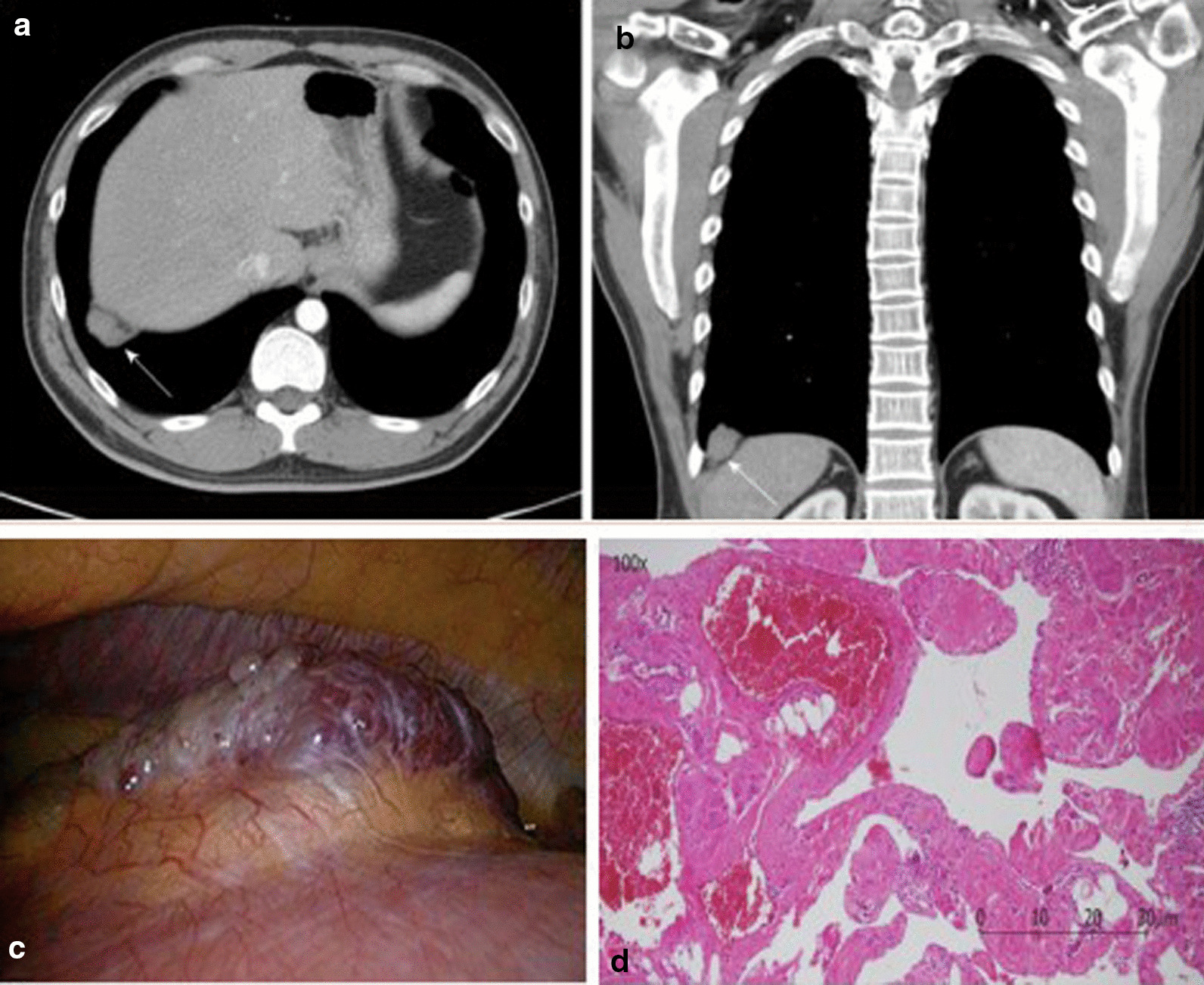
Primary diaphragmatic hemangioma. **a**, **b** Contrast enhanced chest computed tomography images illustrating a poorly-enhanced lesion (white arrow) in the right basal lung, adjacent to the right hemidiaphragm (**a** axial view; **b** coronal view). **c** Intraoperative image of a red papillary tumor arising from the right hemidiaphragm (resected tumor measured 3.5 cm × 1.3 cm). **d** Pathologic image showing a vascular lesion characterized by dilated cavernous vascular space separated by irregular vascular walls microscopically. Pathologic findings are compatible with those of cavernous hemangioma. Reproduced under Open Access Creative Commons License: Chu et al. World J Clin Cases 2019; 7(24):4307

Plain chest radiographs can occasionally be helpful in identifying the etiology of diaphragm dysfunction such as in patients with neoplastic processes in the mediastinum causing phrenic nerve damage (Fig. 28). To assess for subdiaphragm pathology contributing to diaphragmatic dysfunction, CT imaging is a useful tool. Subdiaphragmatic abscess or mass, pancreatitis, ascites, organomegaly or ileus can all affect diaphragm function [1, 116] (Figs. 29, 30). CT may be useful in the identification of contributory pulmonary pathology, such as obstructive lung disease, subpulmonic effusion or pleural thickening [8]. Similar considerations apply to MRI and ultrasound (Fig. 25).

#### Selection of patients for surgical plication

Patients with hemidiaphragm paralysis and eventration who complain of dyspnea [6] and demonstrate hemidiaphragm paradoxical motion and mediastinal shifts may benefit from surgical plication [117, 118]. In contrast, symptomatic patients with an immobile diaphragm do not benefit from surgical plication [119]. While paradoxical motion is thought to represent complete diaphragm denervation, immobility of the diaphragm is thought to result from incomplete denervation injury.

Le Pimpec-Barthes et al. [6] recently reported their experience with the use of dynamic MRI in the surgical treatment of 18 adult patients with unilateral diaphragm eventration.  The investigators concluded that dynamic MRI improves pre-operative knowledge of the status of the hemidiaphragm and permits evaluation of  the quality of surgical repair. In the study, improvement in dynamic MRI correlated with postoperative improvements of dyspnea score and vital capacity.

To summarize, in the clinical setting, the diagnosis of diaphragm paralysis and eventration hinges on detailed imaging of the diaphragm obtained with ultrasound, CT or MRI. MRI is likely the preferential imaging modality as it can assist in the selection of patients who may benefit from surgical plication [6].

### Limitations: ultrasound imaging

Ultrasound imaging of the diaphragm is highly operator-dependent [10] and position-dependent [26]. Operator experience is critical for meaningful image acquisition and interpretation [120]. The variation in repeated measurements either within observers (repeatability) or between observers (reproducibility) can vary up to 18% and 17%, respectively [28, 34]. Failure to properly visualize a hemidiaphragm can be as high as 10% in ambulatory patients [34, 35] and 15% in ICU patients [28, 34]. This is often the result of the downward lung excursion or the limited acoustic window, particularly on the left because of the small dimension of the spleen.  In most adults it is impossible to visualize the entire diaphragm using current ultrasound imaging technology. Whether intrinsic PEEP interferes with the accuracy of ultrasound measurements remains to be determined.

Paradoxical movements of a non-paralyzed diaphragm can occur with hydrothorax, negative pressure pneumothorax, lung fibrosis, atelectasis, and subphrenic abscess [15]. Paradoxical movements of a non-paralyzed diaphragm can also occur with large pleural effusions [121] (Additional files 10 and 11). Maximal voluntary inspiratory maneuvers, required for the measurement of maximal diaphragm excursion, are effort dependent. As a result, any attempt to establish normative data for healthy subjects and for patients with pulmonary or neuromuscular disease, is limited [15]. In addition, inspired lung volumes and diaphragm excursions in patients with lung disease are poorly correlated [50, 122]. This finding reflects the myriad of factors that affect the relative contribution of the diaphragm and other inspiratory muscles to tidal breathing. Body position, weight, height, physical condition and underlying lung pathology all contribute to activation of upper rib cage and neck muscles. The measurement of diaphragm parameters depends critically on lung volume and timing of the respiratory cycle. To standardize how normative data is presented, ultrasound measurement and spirometry should be performed simultaneously [45, 113].

### Limitations: non-ultrasound imaging

Non-ultrasound imaging techniques available to assess diaphragmatic dysfunction have technique-specific limitations. These include lack of specificity (plain radiographs and fluoroscopy), indirect assessment of muscle motion with static images (chest radiograph and CT scan), radiation exposure (plain radiographs, fluoroscopy, CT), bulky equipment and cost (CT, MRI). In addition, limitations that specifically apply to MRI include the impossibility to acquire images in patients with metal implants, and the difficulty to perform the test in patients requiring invasive ventilation or experiencing claustrophobia. Several non-ultrasound imaging techniques, however, are less operator-dependent than ultrasonography of the diaphragm [10]. Moreover, they allow visualization of the entire diaphragm, which is impossible with ultrasonography in adult individuals. How MRI and CT compare to ultrasound imaging for the diagnosis of diaphragm dysfunction is unknown.

### Future directions

Understanding the role of the respiratory muscles in the broader context of pulmonary function is an ongoing challenge. While diaphragm imaging modalities attempt to meet this challenge, refinement of these techniques is required before its use can be broadly advocated for in clinical practice. Shear wave elastography correlates with diaphragmatic pressure output [123] and may represent a promising area for advancement of ultrasound imaging. MRI imaging of the diaphragm is hindered primarily by the need for transport which can be prohibitive for patients in the ICU setting. With advances in portable MRI technology [124], diaphragm MRI in the ICU setting may be possible in the future. Finally, as tidal volume is generated by both the diaphragm and by the extradiaphragmatic muscles, concurrent ultrasound assessment of the diaphragm and the extradiaphragmatic muscles—in particular the parasternal intercostal muscles—could be of interest [125–127]. In this regard, the standard placement of the probe for parasternal intercostal muscle imaging (between the second and third ribs), combined with the location of this muscle group near the surface of the chest wall, may afford generalizability of assessment of this muscle group across research studies [126].

## Conclusions

Knowing the strengths and limitations of imaging techniques of the diaphragm is a critical first step for the evaluation of patients suspected of having diaphragm dysfunction. Diaphragm ultrasound imaging is a cheap and portable technique that allows assessment of diaphragm thickness, thickening, and excursion at a point in time or over time, in ambulatory patients and in mechanically ventilated patients. This technique can also assist in identifying diseases processes responsible for diaphragm dysfunction and in clinical decision making. Qualitative information gathered with ultrasound imaging of the diaphragm about the muscle’s shape, and inspiratory-associated movement and changes in dimensions is, for the most part, solid. The same cannot be stated for quantitative information. The large range of normal values, the operator-dependent nature of image acquisition, and the modest [28] to absent [36] association between ultrasound imaging and diaphragm force output are some of the factors that mar quantitative analysis of ultrasound imaging of the diaphragm. Non-ultrasound imaging techniques can be employed in the evaluation of patients suspected of having diaphragmatic dysfunction. CT imaging and static MRI provide important morphological information about the diaphragm and surrounding structures. Neither, however, offers functional information. The latter can be afforded by conventional fluoroscopy, and more recently, by dynamic MRI. This is an emerging technique that can give information on the contribution of diaphragm dysfunction to pulmonary impairment. The possibility to image the entire thorax and the absence of radiation make dynamic MRI particularly appealing. Despite its limited availability and high cost, dynamic MRI has the potential to be applied in the evaluation patients with neuromuscular diseases [66, 128], and may help in predicting the natural history of respiratory failure in these conditions.

## Supplementary Information

**Additional file 1**. Ultrasound zone of apposition. (*Left panel*) In brightness-mode (B-mode), the diaphragm appears as a three-layer structure: two echogenic layers of peritoneum and pleura sandwiching a more hypoechoic layer of the muscle itself. Occasionally, an additional bright layer which is due to connective tissue and vessels can be seen within the hypoechoic diaphragm muscle layer. (*Right panel*) To identify the diaphragm, subjects are asked to inhale. As the lung comes between the transducer and the diaphragm, it creates a hyperechoic bright artifact (“lung curtain sign”) that obliterates the image of the muscle.

**Additional file 2**. Speckle-tracking analysis of diaphragm contraction. (*Left panel*) The operator traces the inner surface of the two hyperechogenic lines bordering the diaphragm muscle. The analyzed area during the inspiratory effort is the central portion of the region depicted in blue (region of interest). The speckle tracking software follows unique groups of grey-scale pixels (known as ‘kernels’) and measures their displacement and how different ‘kernels’ move in relation to one another (deformation). (*Right panel*) During a diaphragmatic contraction the ‘kernels’ come closer together. The degree of this deformation is known as ‘strain’ and negative values indicate ‘kernels’ coming closer together (Reproduced under Open Access Creative Commons License: Orde et al. BMC Anesthesiol 2015;16(1):43).

**Additional file 3**. Ultrasound shear wave elastography of the diaphragm during inspiratory threshold loading set at 30% of maximal inspiratory pressure. (Upper panel) Diaphragm shear modulus (SMdi) map in kPa measured using shear wave elastography overlaid with standard B-Mode. As the diaphragm contracts and becomes stiffer, the SMdi increases (from purple becomes green and yellow). (Lower panel) Changes in esophageal (Pes), gastric (Pga), and transdiaphragmatic (Pdi) pressures in real time along with ultrasound imaging (Reproduced with permission from The American Physiological Society: Bachasson et al. J Appl Physiol 2019;126:699–707).

**Additional file 4**. Ultrasound dome of the diaphragm. (*Left panel*) Dome of the diaphragm in brightness-mode (B-mode). With this technique, the diaphragm appears as a single thick echogenic line. (*Right panel*) As the diaphragm contracts, the dome moves towards the ultrasound probe.

**Additional file 5**. Normal fluoroscopic sniff test. The test was performed in the anterioposterior and lateral projections, showing normal orthograde motion of both hemidiaphragms during quiet breathing and upon sniff maneuver. (Reproduced with permission from Radiologic Society of North America: Nason et al. Radiographics 2012;32(2):E51–E70).

**Additional file 6**. Fluoroscopic sniff test in a patient with right hemidiaphragm paralysis. Performance of the sniff test in anteroposterior and lateral projections shows paradoxical movements of the right hemidiaphragm. (Reproduced under Open Access Creative Commons License: Kokatnur et al. Diseases 2018;6(1):16).

**Additional file 7**. Fluoroscopic sniff test in a patient with bilateral hemidiaphragm paralysis. During quiet breathing, the test shows no orthograde motion of either hemidiaphragm. On deep breathing, there is paradoxical motion of the right and left hemidiaphragms, which move cranially on inspiration. (Reproduced with permission from Radiologic Society of North America: Nason et al. Radiographics 2012;32(2):E51–E70).

**Additional file 8**. Magnetic resonance imaging fluoroscopy performed during quiet breathing. The test shows physiologic diaphragm movements. (Reproduced with permission from Scientific Scholar: Cicero G et al. J Clin Imaging Sci 2020;10:1.https://doi.org/10.25259/JCIS_138_2019).

**Additional file 9**. Fluoroscopic sniff test; diaphragmatic eventration. The sniff test shows elevation of the anterior aspect of the right hemidiaphragm. During quiet breathing, the right hemidiaphragm has a reduced orthograde motion; on sniffing, there is minimal focal paradoxical motion, which can be seen on the lateral projection.  In contrast, the left hemidiaphragm moves normally. (Reproduced with permission from Radiologic Society of North America: Nason et al. Radiographics 2012;32(2):E51–E70).

**Additional file 10**. Pleural effusion and diaphragm paradox. (*Left panel*) Pre-thoracentesis ultrasound image showing inversion of the left hemidiaphragm and large amount of pleural fluid. (*Right panel*) During inhalation the hemidiaphragm is pulled into the chest cavity while the chest wall expands (paradox). (Reproduced with permission from the American Thoracic Society: Smith et al. Ann Am Thorac Soc 2014;11(8):1323–1326).

**Additional file 11**. Pleural effusion and diaphragm paradox. (*Left panel*) Post-thoracentesis ultrasound image after draining 900 ml of pleural fluid (same patient as in Video 10). The left hemidiaphragm is convex, and the amount of fluid has decreased. (*Right panel*) The hemidiaphragm has regained its normal contour and movement. (Reproduced with permission from the American Thoracic Society: Smith et al. Ann Am Thorac Soc 2014; 11(8):1323–1326).

## Data Availability

Not applicable.

## Abbreviations

CT: Computed tomography
MRI: Magnetic resonance imaging
FRC: Functional residual capacity
LLN: Lower limit of normal
ICU: Intensive care unit
Paw: Airway pressure
Pdi: Transdiaphragmatic pressure
EAdi: Electrical activity of the diaphragm
SM: Shear modulus
SMdi: Diaphragm’s shear modulus
COPD: Chronic obstructive pulmonary disease
FEV_1_: Forced expiratory flow in one second
PawTw: Airway twitch pressure
RV: Residual volume
TLC: Total lung capacity
FVC: Forced vital capacity
VC: Vital capacity
PTPdi: Pressure time product of the diaphragm
Pimax: Maximum effort inspiratory mouth pressure
